# Understanding the impact of correlation within pair‐bonds on Cormack–Jolly–Seber models

**DOI:** 10.1002/ece3.7329

**Published:** 2021-05-01

**Authors:** Alexandru M. Draghici, Wendell O. Challenger, Simon J. Bonner

**Affiliations:** ^1^ Department of Statistical and Actuarial Sciences Western University London Ontario Canada; ^2^ LGL Limited Sidney British Colombia Canada

**Keywords:** Cormack–Jolly–Seber models, correlated fates, goodness‐of‐fit testing, nested models, overdispersion, pair‐bonds, variance inflation factors

## Abstract

1. The Cormack–Jolly–Seber (CJS) model and its extensions have been widely applied to the study of animal survival rates in open populations. The model assumes that individuals within the population of interest have independent fates. It is, however, highly unlikely that a pair of animals which have formed a long‐term pairing have dissociated fates.
2. We examine a model extension which allows animals who have formed a pair‐bond to have correlated survival and recapture fates. Using the proposed extension to generate data, we conduct a simulation study exploring the impact that correlated fate data has on inference from the CJS model. We compute Monte Carlo estimates for the bias, range, and standard errors of the parameters of the CJS model for data with varying degrees of survival correlation between mates. Furthermore, we study the likelihood ratio test of sex effects within the CJS model by simulating densities of the deviance. Finally, we estimate the variance inflation factor $\hat{c}$ for CJS models that incorporate sex‐specific heterogeneity.
3. Our study shows that correlated fates between mated animals may result in underestimated standard errors for parsimonious models, significantly deflated likelihood ratio test statistics, and underestimated values of $\hat{c}$ for models taking sex‐specific effects into account.
4. Underestimated standard errors can result in lowered coverage of confidence intervals. Moreover, deflated test statistics will provide overly conservative test results. Finally, underestimated variance inflation factors can lead researchers to make incorrect conclusions about the level of extra‐binomial variation present in their data.

## Graphical abstract

We present an extension to the Cormack–Jolly–Seber (CJS) model that allows animals who have formed a pair‐bond to have correlated survival and recapture fates. Using the proposed extension to generate data, we conduct a simulation study exploring the impact that correlated fate data has on inference from the CJS model. Our study shows that correlated fates between mated animals may result in underestimated standard errors for parsimonious models, significantly deflated likelihood ratio test statistics, and underestimated values of ĉ for models taking sex‐specific effects into account.

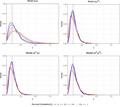

## 1 INTRODUCTION

Mark–recapture experiments are a well‐known and effective method of studying the demographics of wildlife populations (Burnham et al., 1987; King, 2014; King et al., 2009; McCrea, 2014; Seber & Schofield, 2019). Mark–recapture data are collected by capturing individuals from the population at several repeated sampling occasions, marking them with a unique identifier, recording their encounter history, and then releasing them back into the study region (see McCrea, 2014; Seber & Schofield, 2019). The data collected from these studies are typically analyzed by fitting capture–recapture models to generate estimates of the demographic rates pertaining to the open population under study (see Burnham et al., 1987; King, 2014; King et al., 2009; McCrea, 2014; Seber & Schofield, 2019). Most open population models fall within the framework of the Cormack–Jolly–Seber (CJS) model (Cormack, 1964; Jolly, 1965; Seber, 1965). The key assumptions of the CJS model are that survival and recapture fates at any point in the study are constant between animals, all marked animals are correctly recorded, capture–release events are instantaneous (or approximately so), emigration from the sampling region is permanent, and fates of animals are independent of one another (see Seber & Schofield, 2019). Data collected from populations of animals that exhibit complex behaviors are often in violation of the original assumptions of the CJS model. Extensions intended to relax the assumption of constant survival and recapture fates among animals include accounting for heterogeneity with individual‐specific covariates (Gimenez & Barbraud, 2017; Lebreton et al., 1992; Pledger et al., 2003; Royle, 2008), multiple strata (Arnason, 1973), missing covariates (Bonner & Schwarz, 2006), and random effects (see, e.g., Pledger et al., 2003; Royle, 2008). However, nearly all capture–recapture models assume that fates of animals are independent during the sampling period (consider Anderson et al., 1994; Bischof et al., 2020; King, 2014; Lebreton et al., 1992; McCrea, 2014; Seber & Schofield, 2019).

Long‐term pair‐bonds are common among avian species in which a portion of the life‐history pattern is shared between mates (see, for instance, Culina et al., 2013; Maness & Anderson, 2008; Rebke et al., 2017). It is likely that there is correlation between survival or recapture fates for the individuals within a pair (Anderson et al., 1994; Lebreton et al., 1992). Consider, for instance, a motivating example of Harlequin ducks (*Histrionicus histrionicus*), which are waterfowl that typically mate for life (Smith et al., 1996). These ducks migrate from their wintering ground to their breeding grounds with their partners and mostly stay together during the breeding season (Smith et al., 1996). Male Harlequin ducks within a pair‐bond have been shown to be extra‐vigilant in monitoring their nesting partner, which has been theorized to improve survival likelihoods of the female (Bond et al., 2009). Furthermore, a study designed to monitor a population that forms pair‐bonds would likely be performed at the breeding ground due to ease of access. As a consequence, the probability of capturing both individuals within a pair will likely be elevated due to being in close proximity of one another (Lebreton et al., 1992). That said, in some cases, the opposite may be true. For instance, if the male of a pair‐bond is foraging nearby, they may flee when they observe their nesting mate get captured by a research team gathering mark–recapture data. Given the following point, it is reasonable to suspect that the recapture fates of paired individuals may be either negatively or positively correlated. The shared life history and elevated probability of paired individuals constitutes a violation of the standard assumption of independence within capture–recapture models that do not separate their demographic parameters by sex.

Many animals are known to form complex social structures that go beyond that of a pair‐bond. Lowland gorillas, for instance, form harems with one silver‐back male and several females (Hagemann et al., 2019). Another highly social vertebrate is the sperm whale, a mammal that can form multilevel social structures based on smaller long‐term groups called social units (Konrad et al., 2018). Social units are comprised of either a female and younger whales (typically offspring), or a group of mature males (Konrad et al., 2018). As a final example, Dungan et al. (2016) showed that the social alignment of Indo‐Pacific humpback dolphins, a small and isolated population, is centralized around mother–calf rearing groups and that they form both long‐term (years) and short‐term (hours‐days) social associations. As such, failing to account for dependence within populations that contain long‐term social groupings may result in overestimation of the true precision for parameter estimates of common mark–recapture models (see any of Anderson et al., 1994; Bischof et al., 2020; Lebreton et al., 1992).

In this work, we conduct a simulation study to examine the effects that dependence between mated pairs has on inference from the CJS model. Motivated by a long‐term mark–recapture study of Harlequin ducks at the McLeod River region in Alberta, Canada, Challenger (2010) proposed an extension to the CJS framework by introducing a correlation parameter, ρ, to account for the dependence in the recapture events within pairs. Using the work done in Challenger (2010) as the basis for our proposed extension to the CJS model, we introduce another correlation parameter, γ, that accounts for dependence in survival events of pair‐bonded animals. Furthermore, we also allow all pairs to undergo periods of temporary separation when they choose not to breed due to, for instance, external stressors such as lack of food or increased predation (see, e.g., Ludwig & Becker, 2008). During a period of temporary separation, our model treats individuals within a pair as having independent survival and recapture events.

In our simulation study, we assess the standard CJS model's ability to compute accurate demographic estimates for varying levels of survival correlation between mates. Using our proposed extension to generate correlated mark–recapture data, we compute estimates from the standard CJS model and consider the bias, precision, and width of the confidence intervals as survival correlation between pairs increases. Furthermore, our study considered whether asymptotic assumptions of the likelihood ratio test hold when comparing group‐specific CJS models against reduced CJS models in the presence of mated correlation. Finally, we assess the ability of the variance correction $\hat{c}$ (Lebreton et al., 1992) to detect and address the issue of overdispersion due to dependent fates among mated pairs.

## 2 MATERIALS AND METHODS

### 2.1 Model definition

Instead of monitoring all n individuals within a mark–recapture dataset, we instead will consider a collection of n/2≤m≤n entities. An entity j∈{1,⋯,m} is either a set of two animals, male and female, that have formed a pair‐bond or a single animal that has not formed a pair‐bond (originally discussed in Challenger, 2010). We assume that the recapture and survival fates are independent between entities and that individuals within a pair‐bond are strictly monogamous (Challenger, 2010). Furthermore, if an individual within a pairing perishes, at some discrete sampling occasion t∈{1,⋯,T}, in which T is the total number of occasions, then the widowed partner will not seek out a mate during the remainder of the study period (Challenger, 2010). Finally, we condition on the first capture of either individual in an entity in a manner similar to the standard CJS model. When conditioning on the first capture for a pair‐bond, the individuals within the pairing are assumed to have become mates before entering the study (Challenger, 2010).

For the following subsections, consider some fixed entity j∈{1,⋯,m} at some sampling occasion t∈{1,⋯,T}.

#### 2.1.1 Temporary separation process

Let the indicator variable $d_{j,t‐1}∼\mathrm{Bernoulli}(\delta_{j,t‐1})$ denote the event that pair j remain together from time t‐1 to t and $\delta_{j,t‐1}=P\left(d_{j,t‐1}=1\right)$. If a paired entity is temporarily separated, then it is assumed that its member's fates are independent from one another between the sampling periods t‐1 to t. This process occurs before the survival and recapture step at every sampling occasion. Finally, note that if entity j consists of a single individual (widowed or unmated), then $d_{j,t‐1}=0$.

#### 2.1.2 Survival process

In the standard CJS model, it is assumed that the time‐dependent survival process is governed by a Bernoulli distribution, conditioned on the previous survival state (Lebreton et al., 1992). Let $Y_{i,t}|Y_{i,t‐1}∼\mathrm{Bernoulli}(\phi_{i,t‐1}Y_{i,t‐1})$ be the event that individual i∈{1,⋯,n} both survived and remained in the study area from time t‐1 to t. The probability of surviving from t‐1 to t, given that the individual is alive and present at t‐1, is $\phi_{i,t‐1}$. If the individual is dead or has emigrated at time t‐1, they remain so at subsequent time points.

For this extension, we assume that males and females may have distinct probabilities of survival from time t‐1 to t. Let $\phi_{j,t‐1}^{G}$ be the probability that the individual of sex G∈{M,F} of entity j∈{1,⋯,m} survives from time t‐1 to t. For pair‐bonded entities, there are four different survival states in the model: Both members survive, only the female survives, only the male survives, or neither survive (Challenger, 2010). This is represented in the state vector $Y_{j,t}=\left(Y_{j,t}^{M}Y_{j,t}^{F},Y_{j,t}^{F}\left(1‐Y_{j,t}^{M}\right),Y_{j,t}^{M}\left(1‐Y_{j,t}^{F}\right),\left(1‐Y_{j,t}^{M}\right)\left(1‐Y_{j,t}^{F}\right)\right)$ indicating the possible survival outcomes for entity j at time t, in which $Y_{j,t}^{M}$ is the indicator that the male of entity j is alive at time t and $Y_{j,t}^{F}$ is similarly defined for the female of pair j. If both partners are alive at t‐1, then the distribution of $Y_{j,t}$ is governed by a joint Bernoulli distribution with dependent variables (see Appendix A1 for the derivation). The parameters of this distribution are as follows:

- $\Phi_{j,t‐1}^{\mathrm{mf}}=d_{j,t‐1}\gamma_{j,t‐1}\sigma_{\phi ,j,t‐1}^{F}\sigma_{\phi ,j,t‐1}^{M}+\phi_{j,t‐1}^{F}\phi_{j,t‐1}^{M}$ is the probability that both members of entity j survive from t‐1 to t
- $\Phi_{j,t‐1}^{G0}=\phi_{j,t‐1}^{G}‐\Phi_{j,t‐1}^{\mathrm{mf}}$ is the probability that only the individual of sex G∈{M,F} survives from t‐1 to t given that both members were alive at time t‐1
- $\Phi_{j,t‐1}^{00}=1‐\Phi_{j,t‐1}^{\mathrm{mf}}‐\Phi_{j,t‐1}^{m0}‐\Phi_{j,t‐1}^{f0}$ is the probability that both members of entity j perish between times t‐1 to t

where,

- $\sigma_{\phi ,j,t‐1}^{G}=\sqrt{\phi_{j,t‐1}^{G}\left(1‐\phi_{j,t‐1}^{G}\right)}$ is the standard deviation of survival event for individual of sex G∈{M,F} in entity j at time t‐1
- $\gamma_{j,t‐1}\in \left[‐\min\left(\frac{1}{\sqrt{\mathrm{OP}(\phi_{j,t‐1}^{F},\phi_{j,t‐1}^{M})}},\sqrt{\mathrm{OP}(\phi_{j,t‐1}^{F},\phi_{j,t‐1}^{M})}\right),\min\left(\frac{1}{\sqrt{\mathrm{OR}(\phi_{j,t‐1}^{F},\phi_{j,t‐1}^{M})}},\sqrt{\mathrm{OR}(\phi_{j,t‐1}^{F},\phi_{j,t‐1}^{M})}\right)\right]$ is the correlation coefficient for survival of pair j from t‐1 to t (see Appendix A2 for the derivation of the bounds and definitions of the odds ratio (OR) and the odds product (OP)).

Finally, we condition on $d_{j,t‐1}$ such that if there is temporary separation, then the correlation coefficient becomes zero and $Y_{j,t}$ becomes the product of two independent Bernoulli variables. Now the partially observed survival process for entity j at time t can be described with the following multinomial distribution:

(1) $$Y_{j,t}|Y_{j,t-1},d_{j,t-1}∼\mathrm{Multi}\left(1,Y_{j,t-1}\left[\begin{array}{cccc} \Phi_{j,t-1}^{\mathrm{mf}} & \Phi_{j,t-1}^{f0} & \Phi_{j,t-1}^{m0} & \Phi_{j,t-1}^{00}\\ 0 & \phi_{j,t-1}^{F} & 0 & 1-\phi_{j,t-1}^{F}\\ 0 & 0 & \phi_{j,t-1}^{M} & 1-\phi_{j,t-1}^{M}\\ 0 & 0 & 0 & 1 \end{array}\right]\right).$$

#### 2.1.3 Recapture process

Consider the standard CJS model, we assume that the observation process is governed by a Bernoulli distribution conditioned on the current survival state (Lebreton et al., 1992). Let $X_{i,t}|Y_{i,t}∼\mathrm{Bernoulli}(p_{i,t}Y_{i,t})$ be the event that individual i∈{1,⋯,n} was recaptured at time t. The probability of being recaptured at time t, given that the individual is alive and present at t, is $p_{i,t}$.

For this extension, we assume that males and females may have distinct recapture probabilities at time t. Let $p_{j,t}^{G}$ be the probability that the individual of sex G∈{M,F} of entity j∈{1,⋯,m} is recaptured at time t. There are four different recapture outcomes for paired entities in the model: Both members are observed, only the female is observed, only the male is observed, or neither are observed (Challenger, 2010). The possible recapture outcomes for entity j at time t can be represented by the vector $X_{j,t}=\left(X_{j,t}^{M}X_{j,t}^{F},X_{j,t}^{F}\left(1‐X_{j,t}^{M}\right),X_{j,t}^{M}\left(1‐X_{j,t}^{F}\right),\left(1‐X_{j,t}^{M}\right)\left(1‐X_{j,t}^{F}\right)\right)$, in which $X_{j,t}^{M}$ is the indicator that the male of entity j is recaptured at time t and $X_{j,t}^{F}$ is analogously for the female. If both partners are alive, then the distribution of $X_{j,t}$ is governed by a joint Bernoulli distribution with dependent variables (see Appendix A1 for the derivation). The parameters of this distribution are as follows:

- $P_{j,t}^{\mathrm{mf}}=d_{j,t‐1}\rho_{j,t}\sigma_{p,j,t}^{F}\sigma_{p,j,t}^{M}+p_{j,t}^{F}p_{j,t}^{M}$ is the probability that both members in pair j are captured at time t
- $P_{j,t}^{G0}=p_{j,t}^{G}‐P_{j,t}^{\mathrm{mf}}$ is the probability that only the individual of sex G∈{M,F} is captured at time t, given that both members were alive at time t
- $P_{j,t}^{00}=1‐P_{j,t}^{\mathrm{mf}}‐P_{j,t}^{m0}‐P_{j,t}^{f0}$ is the probability that both members of pair j are unobserved at time t

where,

- $\sigma_{p,j,t}^{G}=\sqrt{p_{j,t}^{G}\left(1‐p_{j,t}^{G}\right)}$ is the standard deviation of recapture for individual of sex G∈{M,F} in entity j at time t
- $\rho_{j,t}\in \left[‐\min\left(\frac{1}{\sqrt{\mathrm{OP}(p_{j,t}^{F},p_{j,t}^{M})}},\sqrt{\mathrm{OP}(p_{j,t}^{F},p_{j,t}^{M})}\right),\min\left(\frac{1}{\sqrt{\mathrm{OR}(p_{j,t}^{F},p_{j,t}^{M})}},\sqrt{\mathrm{OR}(p_{j,t}^{F},p_{j,t}^{M})}\right)\right]$ is the correlation coefficient for recapture between members of pair j at time t.

Finally, we condition on $d_{j,t‐1}$ such that if there is temporary separation, then the correlation coefficient becomes zero and $X_{j,t}$ becomes the product of two independent Bernoulli variables. Now the recapture process for entity j at time t can be described with the following multinomial distribution:

(2) $$X_{j,t}|Y_{j,t},d_{j,t-1}∼\mathrm{Multi}\left(1,Y_{j,t}\left[\begin{array}{cccc} P_{j,t}^{\mathrm{mf}} & P_{j,t}^{f0} & P_{j,t}^{m0} & P_{j,t}^{00}\\ 0 & p_{j,t}^{F} & 0 & 1-p_{j,t}^{F}\\ 0 & 0 & p_{j,t}^{M} & 1-p_{j,t}^{M}\\ 0 & 0 & 0 & 1 \end{array}\right]\right)$$

### 2.2 Simulation study

#### 2.2.1 Data generating process

To study the impact of dependence between mated individuals on the standard CJS model, we used the statistical programming software R (R Core Team, 2020) to generate 1000 samples from the extended model (detailed in Section 2.1) for each of the following parameter settings:

- n=200 (Fixed Sample Size)
- T=4 (Fixed Number of Sampling Occasions)
- $\delta_{j,t}=1.0$ (Fixed Probability of Remaining Together for Mated Pairs)
- $\phi_{j,t}^{F}=\phi_{j,t}^{M}=0.7$ (Fixed Survival Probabilities)
- $p_{j,t}^{F}=p_{j,t}^{M}=0.8$ (Fixed Recapture Probabilities)
- $\gamma_{j,t}\in \left\{‐0.4,‐0.3,\cdots ,0.9,1.0\right\}$ (Grid of Survival Correlations)
- $\rho_{j,t}\in \left\{‐0.25,0.0,0.25,0.5,1.0\right\}$ (Grid of Recapture Correlations)

in which these settings hold ∀j∈{1,⋯,m} and t∈{1,⋯,T}. Moreover, we simulated the sex of each animal with an unbiased coin toss. We assumed that all 200 individuals were marked on the first occasion (a single cohort) and that there are as many pairings as possible. Specifically, if there were 105 simulated males and 95 females there would be 95 mated pairs, 10 unmated males, and a total of m=105 entities in our sample. Finally, we assumed that there was no temporal variation across all parameters. Given this, we omit the subscripts j and t going forward. Note that the case in which γ=0 and ρ=0 is equivalent to the standard CJS model.

#### 2.2.2 Data modeling process

We used the standard CJS model to compute estimates of survival and recapture rates, goodness‐of‐fit statistics, and overdispersion corrections of the data we simulated from the extended model (Section 2.1) using program MARK (White & Burnham, 1999), a popular mark–recapture modeling software among ecological researchers, with the R library RMark (Laake, 2013). We consider the following parameter settings of the standard CJS model:

(3) $$\left\{\left(\phi ,p\right),\left(\phi^{G},p\right),\left(\phi ,p^{G}\right),\left(\phi^{G},p^{G}\right)\right\}$$

in which, using the notation discussed in Burnham et al. (1987), $\phi^{G}$ denotes a sex‐specific effect for survival and $p^{G}$ denotes a sex‐specific effect for recapture. For instance, $\left(\phi^{G},p\right)$ represents the case in which the standard CJS model has a sex‐specific effect for survival probability and a common recapture rate for both sexes.

#### 2.2.3 Standard metrics to assess model performance

To study the impact that varying levels of survival correlation within mark–recapture data has on estimates of survival rates, we computed the range and coverage percentage of the corresponding 95% confidence intervals, along with the relative bias of the survival estimates. The results were computed across a grid of survival correlations ranging from ‐0.4 to 1.0 increasing by increments of 0.1 for model (ϕ,p). Furthermore, we present the percent coverage of the 95% confidence intervals for each of the cases in equation 3. Finally, in order to better isolate the impact of correlation within entities on the hidden state process, we set the recapture correlation between mated pairs to zero.

Let K=1000 denote the number of replicate data sets for each scenario and $\hat{\phi }:=\sum_{k=1}^{K}{\hat{\phi }}_{k}/K$ where ${\hat{\phi }}_{k}$ represents the estimate of ϕ from the $k^{\mathrm{th}}$ replicate. Let ${\mathrm{UB}}_{k}$ and ${\mathrm{LB}}_{k}$ denote the kth values of the upper and lower bounds of the 95% confidence intervals of ${\hat{\phi }}_{k}$, respectively. Our computed simulation study metrics are then:

- Mean Relative Bias: $B\left(\phi \right):=\sum_{k}\left({\hat{\phi }}_{k}‐\phi \right)/K\phi =\left(\hat{\phi }‐\phi \right)/\phi$,
- Mean Relative 95% CI Width: $R\left(\phi \right)=\sum_{k}\left({\mathrm{UB}}_{k}‐{\mathrm{LB}}_{k}\right)/K\phi$,
- Percent Coverage of 95% CI: $C\left(\phi \right)=\sum_{k}I\left(\hat{\phi }\in \left[{\mathrm{LB}}_{k},{\mathrm{UB}}_{k}\right]\right)/K$,

in which I(A) denotes the indicator function of some event A occurring.

#### 2.2.4 The likelihood ratio test in mark–recapture modeling

The likelihood ratio test (LRT) is a statistical test used to compare a general model against a nested model that exists on a reduced parameter space (Anderson et al., 1994; Lebreton et al., 1992). The test determines whether the reduced model captures a sufficient amount of variability relative to the general model (Anderson et al., 1994; Lebreton et al., 1992). Consider a case of the CJS model in which we are testing whether survival varies by sex and we assume that recapture does not. Then, our hypothesis test can be expressed as:

$$H_{0}:\phi^{F}=\phi^{M}\;\;\;\;\&\;\;\;\;p^{F}=p^{M}$$

$$H_{\alpha }:\phi^{F}\neq \phi^{M}\;\;\;\;\&\;\;\;\;p^{F}=p^{M}$$

The likelihood ratio statistic is defined as the ratio between the likelihood maximized over the null hypothesis and the likelihood maximized over alternative (Anderson et al., 1994; Lebreton et al., 1992):

(4) $$\Delta :=\frac{{\mathrm{Sup}}_{\left(\phi ,p\right)}L\left(\phi ,p|y\right)}{{\mathrm{Sup}}_{\left(\phi^{F},\phi^{M},p\right)}L\left(\phi^{F},\phi^{M},p|y\right)}.$$

The test statistic, called the deviance, is then $G^{2}:=‐2\log\left(\Delta \right)$. Under the null hypothesis, the deviance follows the chi‐squared distribution with degrees of freedom equal to the difference between the degrees of freedom between the general and reduced model (Anderson et al., 1994; Lebreton et al., 1992). In our example, we have $G^{2}\overset{H_{0}}{∼}\chi_{1}^{2}$ and our p‐value is then computed with $p=P(X_{1}^{2}\geq G^{2})$ in which $X_{1}^{2}∼\chi_{1}^{2}$. Moreover, by the probability integral transformation theorem, we know that $p\overset{d}{∼}U(0,1)$.

In our study, we compared the probability densities of both the deviance statistic and the corresponding p‐value for the both the LRT comparing $\left(\phi^{G},p\right)$ against (ϕ,p) and $\left(\phi ,p^{G}\right)$ against (ϕ,p) across γ∈{0.0,0.3,0.6,0.9,1.0} with a fixed value of ρ=0.0. We investigated whether dependence between mated pairs in mark–recapture data impacted the ability of the LRT to perform reliable model selection.

#### 2.2.5 The $\hat{c}$ correction in mark–recapture models

When mark–recapture data are thought to violate the model assumption of regular binomial variation, an estimate of the variance inflation factor, called $\hat{c}$, can be computed to assess the level of overdispersion in the model. Under appropriate binomial variation, data that emerged from the CJS model would give a result of $\hat{c}\approx 1$ (Anderson et al., 1994). On the other hand, $\hat{c}>>1$ suggests that the data has excess variation implying that either the model structure is inadequate ($\hat{c}>>5$) or the underlying model assumptions have been violated (Anderson et al., 1994). One well‐known consequence of overdispersion due to the dependent fates of individuals is that standard error estimates will by understated by the CJS model (see Anderson et al., 1994; Bischof et al., 2020). The recommended approach to dealing with this in practice is to scale up the standard error by a factor of $\sqrt{\hat{c}}$ (Anderson et al., 1994; Lebreton et al., 1992; Pradel et al., 2005). Furthermore, Anderson et al. (1994) have shown that the presence of overdispersion due to data replication can impact goodness‐of‐fit testing by inflating the deviance statistic which increases the type I error rate of the LRT.

There are three popular estimators of overdispersion in mark–recapture modeling (Cooch & White, 2020). They can be referred to as the deviance $\hat{c}$ estimator (Anderson et al., 1994), Pearson's (or the chi‐square) $\hat{c}$ estimator (Lebreton et al., 1992; Pradel et al., 2005), and Fletcher's $\hat{c}$ estimator (Fletcher, 2012). In our study, we consider the deviance approach. Specifically, when performing model selection the most general model should fit the data reasonably well compared to the saturated model, otherwise the data are likely to have extra‐binomial variation (Anderson et al., 1994; Lebreton et al., 1992). The deviance between the saturated model and the general model over the difference in their degrees of freedom can be used to compute an approximation to the distribution of the variance inflation factor (Anderson et al., 1994),

(5) $$\hat{c}∼\frac{\chi_{{\mathrm{df}}_{\mathrm{deviance}}}^{2}}{{\mathrm{df}}_{\mathrm{deviance}}}.$$

In our simulation study, we drew samples from the density of $\hat{c}$ and generated a point estimate of the overdispersion by taking the median. We call it the median $\hat{c}$ estimator (similar to the median $\hat{c}$ estimator discussed in Cooch & White, 2020), and it is denoted as ${\hat{c}}_{\mathrm{med}}:=\mathrm{median}\left(\hat{c}\right)$. We repeated this process for different values of γ∈{0.0,0.3,0.6,0.9,1.0} and a fixed ρ=1.0. We assessed whether variation induced by mated pairs having correlated fates is detectable by considering whether the density of $\hat{c}$ and the corresponding point estimates, ${\hat{c}}_{\mathrm{med}}$, indicated overdispersion. In order to assess whether the behavior of the estimator is in line with current literature, we computed ${\hat{c}}_{\mathrm{med}}$ for all four model settings in equation 3.

## 3 RESULTS

### 3.1 Standard errors for CJS models under pair‐specific linear correlation

**FIGURE 1:**
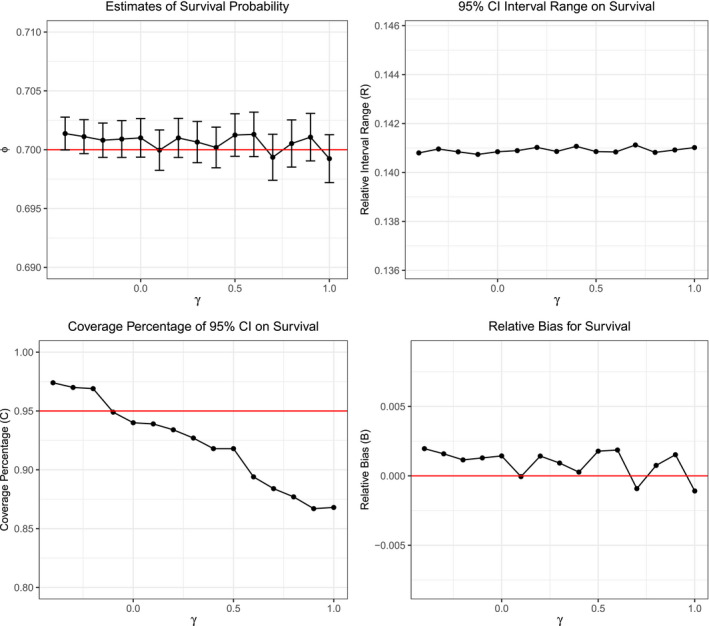
Survival metrics against survival correlation (γ) for (ϕ,p). Top Left: Monte Carlo estimates of survival $\hat{\phi }$ across varying levels of γ. The error bars represent the 95% Monte Carlo confidence intervals, which are approximately equal to $\hat{\phi }\pm 1.96\frac{\sigma }{\sqrt{K}}$. The red line represents the truth ϕ=0.7; Top Right: Interval width of 95% confidence intervals on $\hat{\phi }$ across varying levels of γ; Bottom Left: Coverage percentage of the confidence intervals for $\hat{\phi }$ across varying levels of γ. The red line represents the 95% confidence level; Bottom Right: Relative bias of $\hat{\phi }$ across varying levels of γ. The red line indicates a relative bias of zero

**FIGURE 2:**
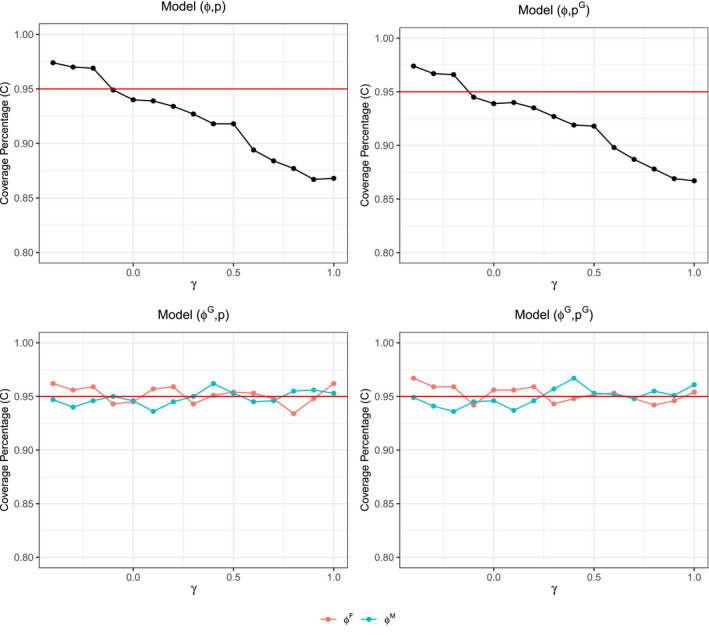
Coverage percentage of the confidence intervals for $\hat{\phi }$ across varying levels of γ for all models $\left\{\left(\phi^{G},p^{G}\right),\left(\phi^{G},p\right),\left(\phi ,p^{G}\right),\left(\phi ,p\right)\right\}$. Red line is 95% confidence level

Monte Carlo estimates for the survival probability, relative confidence interval width, and relative bias in model (ϕ,p) are not impacted by changes in the amount of survival correlation present between mated pairs in the data (see Figure 1). That said, as survival correlation increases between mated pairs, the percent coverage of the confidence intervals decreases below the expected 95% value down to an extreme of about 87% (Figure 1). This implies that the standard errors of the survival probability estimates are being understated by the (ϕ,p) model, since they are the only term that in the confidence bounds that can vary due to the data. Moreover, percentage coverage is only understated at high levels of survival correlation in models that do not account for the effect of sex on survival (see Figure 2). On the other hand, the models that account for sex‐specific differences in their survival probabilities have coverage percentages that tend to stay around 95%, with acceptable statistical variation, and thus continue to produce reliable standard error estimates (Figure 2).

### 3.2 Behavior of the LRT under Pair‐Specific Linear Correlation

**FIGURE 3:**
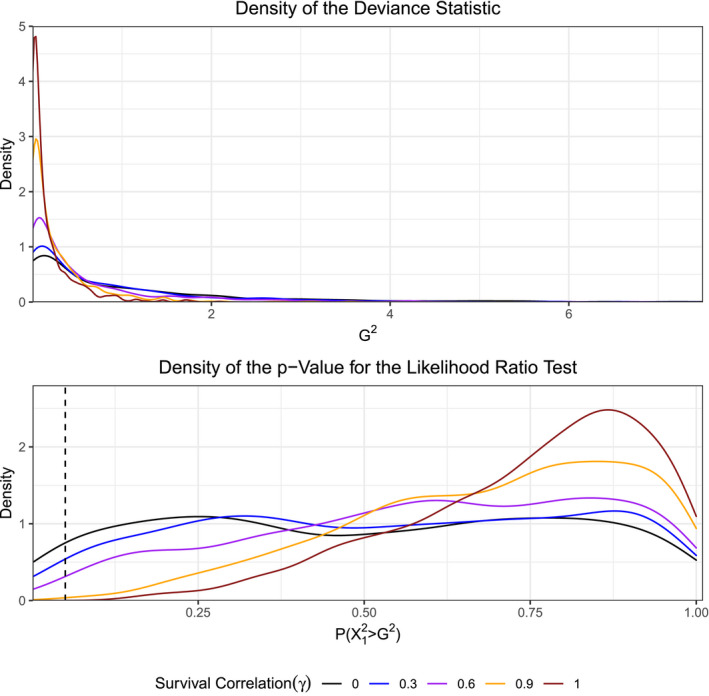
Likelihood ratio test of $\left(\phi^{G},p\right)$ versus (ϕ,p) in which ρ=0 across a grid of survival correlations γ∈{0,0.3,0.6,0.9,1.0}. Dashed line at the value of $P(X_{1}^{2}\geq G^{2})=0.05$

**FIGURE 4:**
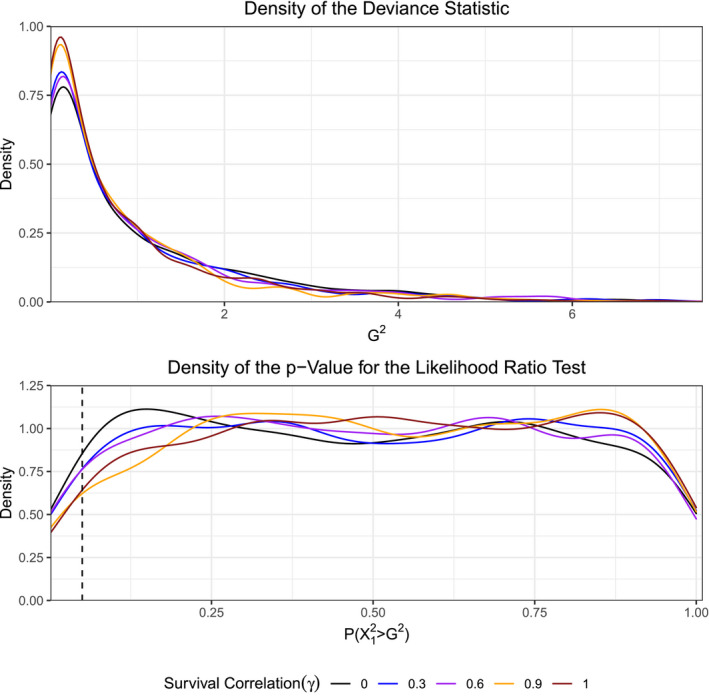
Likelihood ratio test of $\left(\phi ,p^{G}\right)$ versus (ϕ,p) in which ρ=0 across a grid of survival correlations γ∈{0,0.3,0.6,0.9,1.0}. Dashed line at the value of $P(X_{1}^{2}\geq G^{2})=0.05$

As the level of survival correlation within the data increases, the tails of the density for the likelihood ratio test statistic, comparing models $\left(\phi^{G},p\right)$ and (ϕ,p), become lighter than those of the assumed $\chi_{1}^{2}$ distribution (Figure 3). The density of the p‐values, in turn, shift from a uniform distribution toward a left‐skewed one (Figure 3). The case in which there is no survival or recapture correlation serves as a basis of comparison. This result implies that the likelihood ratio test will not reject the underlying null hypothesis with a probability equal to its significance level (in this case α=0.05), but will instead fail‐to‐reject with a higher probability. The violation of the independence assumption across observations deflates the deviance statistic leading to the goodness‐of‐fit test favoring the more parsimonious hypothesis. A technical example illustrating why the density of the deviance begins to shrink toward zero as the survival and recapture correlation increases is available in Appendix B2. Interestingly, if we consider the likelihood ratio test between models $\left(\phi ,p^{G}\right)$ and (ϕ,p) (Figure 4), in which the recapture correlation is fixed at ρ=0, we find that added survival correlation has minimal impact on the test's efficacy. These results suggest that increasing mated survival correlation between paired individuals does not have a large impact on goodness‐of‐fit testing for sex effects in recapture rates. Overall, the goodness‐of‐fit test comparing the effect of sex on survival is impacted by survival correlation between mated pairs, while the test comparing the effect of sex on recapture is not.

### 3.3 Behavior of the $\hat{c}$ correction under pair‐specific linear correlation

**FIGURE 5:**
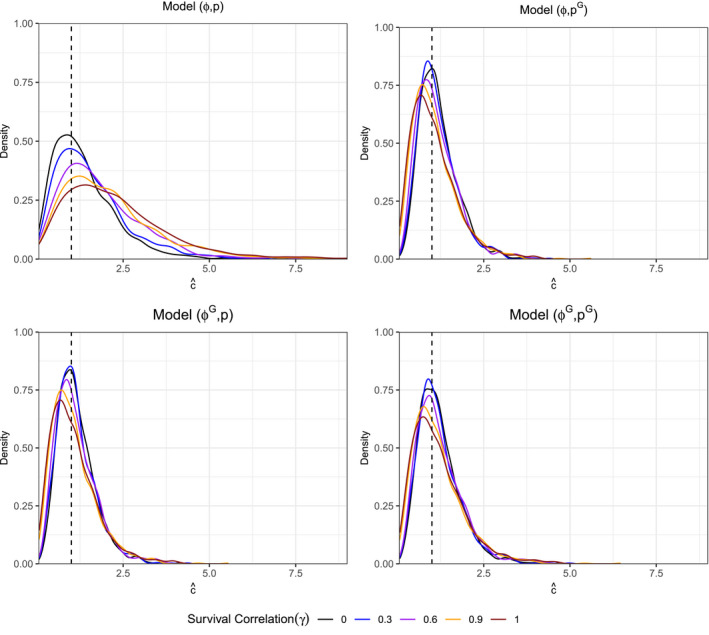
Density of $\hat{c}$ for all models $\left\{\left(\phi^{G},p^{G}\right),\left(\phi^{G},p\right),\left(\phi ,p^{G}\right),\left(\phi ,p\right)\right\}$ in which ρ=1 across γ∈{0,0.3,0.6,0.9,1.0}. Dashed line at the value of $\hat{c}=1$

For models that account for sex in either of their parameter estimates (all but (ϕ,p)), the sampling densities of $\hat{c}$ (see Figure 5) are within a close neighborhood of 1.0, regardless of survival or recapture correlation between mates. In fact, with the exception of (ϕ,p) the median estimate of $\hat{c}$ decreases as the survival correlation increases (see Table 1). For these model settings, $\hat{c}$ has proven incapable of detecting the violated assumption of independence within the data. However, model (ϕ,p) does not account for sex‐specific differences in its parameter estimation and so when γ=1 and ρ=1 the mark–recapture data appear to be nearly replicates. Anderson et al. (1994) showed that under this construction (replicated data without assigning treatment groups to each replicate) ${\hat{c}}_{\mathrm{med}}\approx 2$. (ϕ,p) can be thought of as a control with respect to the other models in the study. Given that estimates of c are typically computed from the most general model under examination (Cooch & White, 2020), the variance correction would not be applied to the standard errors or be used to rescale goodness‐of‐fit testing metrics. As such, when data replication occurs due to correlation among treatment groups (sex in our example), the $\hat{c}$ estimator will be understated for studies that include these groups in their construction.

**TABLE 1:** Median($\hat{c}$) for varying levels of (γ) across all models

Model	Survival Correlation				
	γ=0.0	γ=0.3	γ=0.6	γ=0.9	γ=1.0
(ϕ,p)	1.17	1.34	1.59	1.86	2.00
$\left(\phi ,p^{G}\right)$	1.09	1.06	1.03	0.94	0.93
$\left(\phi^{G},p\right)$	1.05	1.04	1.01	0.93	0.93
$\left(\phi^{G},p^{G}\right)$	1.10	1.09	1.08	1.02	1.03

## 4 DISCUSSION

The results of our study show that the presence of correlation between paired individuals introduces extra‐binomial variation to the data, resulting in underestimated standard errors and lowered coverage of confidence intervals for models that fail to account for sex‐specific effects. Our example in Appendix B1 shows that the most extreme case of paired correlation in the data corresponds to $\hat{c}\approx 2$.

Furthermore, we have identified an issue with the inferences provided by the likelihood ratio test. Sex‐specific correlation in the data caused the asymptotic distribution of the simulated deviance statistic to differ from its theoretical distribution for the test of whether there was an effect of sex present in survival and/or recapture rates. As such, increased levels of correlation for survival and/or recapture outcomes resulted in overly conservative test results (failure to reject $H_{0}$ more frequently than theoretically expected). Issues with asymptotic assumptions surrounding the likelihood ratio test in mark–recapture models are not unique to this study. Sparse contingency tables have been shown to skew the density of the deviance statistic (both up and down) stemming from the likelihoods of multinomial models (Afroz et al., 2019; Koehler, 1986). By introducing correlation into the CJS model structure, we are, in a sense, reducing the effective sample size of each generated dataset. Consider an example in which recapture and survival correlations are set to one in a population of 200 animals consisting of exactly 100 males and females with each animal in a long‐term pair‐bond. Under this setup, each pair effectively acts as a single individual (Lebreton et al., 1992). If one animal from the pair dies (or is recaptured), then its partner will die (or be caught) as well. In this case, we need only model the outcomes of one individual from each pair‐bond using the standard CJS model to compute reliable estimates of the survival and recapture probabilities. This is, in effect, reducing our sample size down from n=200 down to n=100 and halving the expected cell frequencies of our contingency table as well. We contend, however, that sparse data are not the key issue at play here as we designed our simulation study to mitigate these known effects. Recall that the survival and recapture probabilities used to generate our data were 0.7 and 0.8 across all time points for all individuals, respectively. Furthermore, our simulation included one cohort in which all first captures occurred at time t=1. Table 2 shows the expected cell frequencies in our simulation study for the cases in which n=100 and n=200. Koehler and Larntz (1980) showed that the distribution of the deviance is not well approximated by the chi‐squared distribution when the ratio of the sample size against the number of possible cells is less than five. In our case, this ratio is equal to n/8=25 and so we expect that the deviance should be asymptotically chi‐squared. Moreover, if the majority of expected cell frequencies lie below 0.5, then the test is said to be overly conservative (Larntz, 1978). On the other hand, if most of the cell frequencies lie within the interval [0.5,4], then the test becomes too liberal (rejects $H_{0}$ too often) (Koehler, 1986).

**TABLE 2:** Recapture history cell probabilities and expected number of observed histories (for populations with *n* = 100 and *n* = 200 individuals) used in simulation study

Histories	Probability	Expected (*n* = 100)	Expected (*n* = 200)
1000	0.351	35.1	70.1
1011	0.044	4.4	8.8
1101	0.044	4.4	8.8
1110	0.138	13.8	27.6
1100	0.202	20.2	40.5
1010	0.034	3.4	6.9
1001	0.011	1.1	2.2
1111	0.176	17.6	35.1

The expected cell frequencies shown in Table 2 all lie above 0.5 for both n=100 and n=200. While sparsity will have an impact on the distribution of the deviance, the extreme shift from the chi‐squared distribution that we observe goes well beyond the expected difference introduced by sparsity found in our simulated data. The large spike in p‐values as correlation increases is largely due to the nature of the duplicated data along with the models under consideration in our simulation study. Consider Appendix B2 for a mathematical example illustrating why correlation within groups in mark–recapture data deflates the deviance of the likelihood ratio test along with a small simulation study showing the effect of increased sparsity on the density of the deviance statistic without any correlation present between sexes. Furthermore, we acknowledge that in many field studies the recapture rate in are lower than 80%. In these cases, it becomes increasingly difficult to isolate the cause of deviations from the chi‐squared distribution.

Anderson et al. (1994) showed that mark–recapture data with overdispersion due to data replication inflate the size of the deviance when comparing across CJS models that fail to account for the cause of the data replication. Our results show that the source of overdispersion and the models under consideration are vital components to determining the behavior of the deviance. When replicated mark–recapture data are split by treatment groups (males and females) and the mark–recapture model used to study the data accounts for these groups in its parameter estimates, we have shown that the computed values of $\hat{c}$ are understated. This case occurs when comparing group‐specific heterogeneity for data in which there is a significant amount of correlation between the two groups being tested. Therefore, we need to both identify whether there is replication in our sampling data and if there is an underlying group structure separating the replicates (in our example the sex of the animals).

For models that took group‐specific heterogeneity into account, estimates of the overdispersion parameter $\hat{c}$ were too small to indicate any significant departure from binomial variation, regardless of the degree of survival and recapture correlation. As such, overdispersion due to dyadic correlation in populations that are highly segmented into pairs may not be easily detectable. Consider, Appendix B3 for a technical example demonstrating why this is the case. The small study presented in Appendix B3 shows that these results also apply to the Pearson (Pradel et al., 2005) and Fletcher's (Fletcher, 2012) $\hat{c}$ estimators. The overdispersion introduced by our model does not result in a large violation of the inherent structure of the CJS model. The new parameters δ,γ,ρ are, in essence, controlling how similar the male and female sample data will be to one another. The estimates of ϕ and p will remain largely unbiased because the maximum‐likelihood estimation procedure is unaffected by departures in binomial variation (see the discussion in Pradel et al., 2005). Lack of biased estimates is not surprising when dealing with unmodeled dependence structures in mark–recapture data. For instance, Challenger (2010) found that the CJS model produced reasonably unbiased estimates when modeling data with group‐specific correlations using Bayesian methods. Bischof et al. (2020) also showed that spatial capture–recapture models with induced correlation between groups (of sizes ≥2) did not lead to heavily biased estimates of model parameters. As such, if the estimates of c were able to reliably detect overdispersion introduced by high dyadic correlations, quasi‐likelihood approaches should provide a reasonable adjustment to standard error estimates (Anderson et al., 1994). The issue is that the estimator $\hat{c}$ is incapable of reliably detecting overdispersion in replicated data when the replicates are accounted for in the modeling process as groups. Unfortunately, we have shown here that failing to account for correlation between mated pairs has the significant consequence of severely violating the asymptotic assumptions of the likelihood ratio test and understating standard errors in reduced models. Lebreton et al. (1992) suggested that when dealing with highly correlated data between sexes it may be reasonable to consider the sample population of only one sex. Indeed, this approach will mitigate issues of understated standard errors and failings of the variance inflation factor. However, one would need *a priori* knowledge of the dependence between mated pairs in order to make this judgment, as we have shown that the likelihood ratio test for group‐specific differences, sometimes referred to as TEST1 (Burnham et al., 1987), will overly favor the null hypothesis $H_{0}$ for data with high levels of pair‐specific correlation. In an applied setting, researchers will not be able to determine whether the LRT favors the more parsimonious model because of excessive correlation between mated pairs or whether it is due to the parameters of interest being the same between both sexes without any large violations to independence. As such, it is important to be conscious of these issues when studying animal populations that are suspected to form correlated known social groupings. If a researcher suspects this to be the case, we suggest analyzing the data for each sex separately in order to isolate the source of overdispersion. For instance, one can simulate estimates of c using the full data with the model (ϕ,p) (see chapter 5 in Cooch & White, 2020), separate the data by sex, and then repeat the process for each subset of the data. If the majority of the overdispersion stems from group‐specific correlations, the $\hat{c}$ estimates generated from the data for each specific sex should be close to one. If, however, the $\hat{c}$ estimates remain high for each group, then it is likely that there may be other major sources of extra‐binomial variation present within the data. When a large majority of the overdispersion comes from association between known pairs, the researcher should either scale the standard errors and information criteria with the $\hat{c}$ estimate from (ϕ,p) or study the data for only one of the two sexes.

A cleaner approach would be to estimate group‐specific correlation explicitly using extended models. Directly estimating group‐specific correlation with mark–recapture models will allow researchers to glean further insights into the social dynamics at play between individuals within the population of interest. For instance, we could study how the effect sizes of meaningful covariates pertaining to survival rates change in the presence of group‐specific correlations. Does having a mate improve or hamper the chance of an animal surviving when facing external selective pressures? There are, however, a whole new set of issues that come with explicitly modeling group‐specific correlations as well. The assumption of mated pairs forming permanent (even in highly socially monogamous populations) pairings is unrealistic and can lead to issues with parameter estimation (Gimenez et al., 2012). Furthermore, by conditioning on long‐term pair‐bonds already existing we limit the applicability of our proposed model to mature animals, as juveniles cannot be in a long‐term pair before maturity. Divorce is quite common among animals that form long‐term mate pairings (Culina et al., 2013; Gimenez et al., 2012; Ludwig & Becker, 2008; Maness & Anderson, 2008; Smith et al., 1996). Researchers will need to explicitly model the mate status of each individual animal, their current partner, and their partner transitions, otherwise risk issues of pseudo‐replication (Culina et al., 2013). The issue of missing data is inflated here as well—what if one of the study participants is mated with an individual who has not yet been tagged? In most capture–recapture studies, social detection is imperfect, even among animals with highly correlated fates (Gimenez et al., 2019; Hoppitt & Farine, 2018). One might suggest omitting the data points for animals that are seen with multiple partners in populations that mostly practice social monogamy (low divorce rates). Unless the population has very few cases of partner swapping, omitting these individuals will likely result in inflated standard errors and biased estimates. The question then becomes: Should we risk understated or overstated standard errors when modeling our data? Finally, estimating the correlations of demographic parameters between different groups of animals (adult versus juvenile for instance) often requires populations with a large number of marked individuals to achieve a reasonable degree of estimate precision (see Riecke et al., 2019). These issues will need to be addressed in future work if social independence is to be accounted for with an extended and estimable model structure.

## CONFLICT OF INTEREST

The authors have no conflicts of interest to declare.

## AUTHOR CONTRIBUTION

**Alexandru Marian Draghici:** Conceptualization (equal); Data curation (lead); Formal analysis (lead); Funding acquisition (supporting); Investigation (equal); Methodology (equal); Software (lead); Validation (lead); Visualization (equal); Writing—original draft (lead); Writing—review and editing (equal). **Wendell Challenger:** Conceptualization (supporting); Methodology (equal); Validation (equal); Writing—review and editing (supporting). **Simon Bonner:** Conceptualization (equal); Formal analysis (equal); Funding acquisition (lead); Investigation (equal); Methodology (equal); Software (supporting); Validation (equal); Visualization (equal); Writing—original draft (supporting); Writing—review and editing (equal).

## Figures and Tables

**FIGURE 6:**
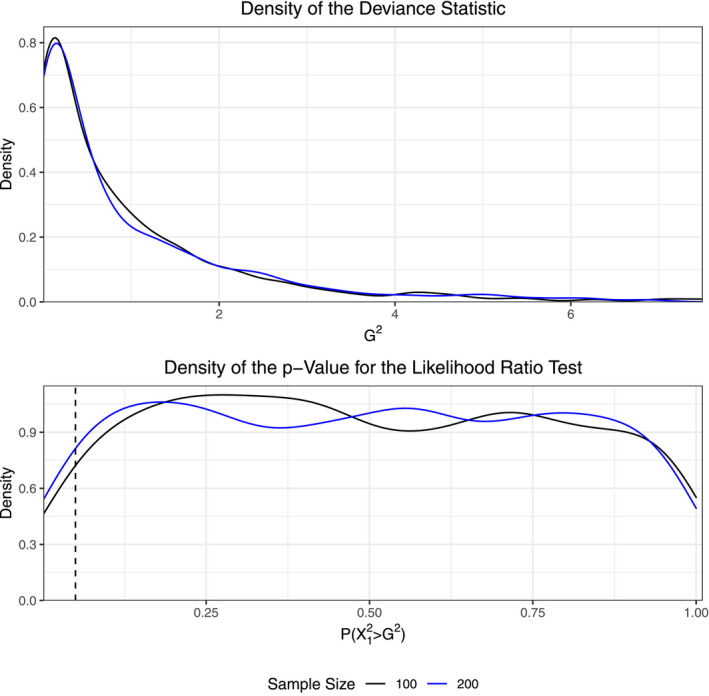
Density of the deviance and the p‐values of the likelihood ratio test for $\left(\phi^{G},p\right)$ versus (ϕ,p) in which ρ=0 and γ=0 for both *n* = 100 and *n* = 200. Dashed line at the value of $P(X_{1}^{2}\geq G^{2})=0.05$

**FIGURE 7:**
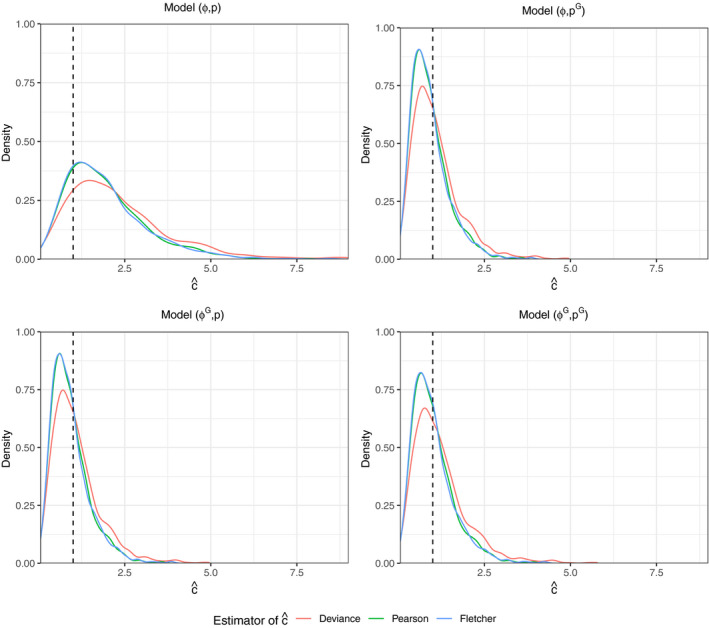
Density of commonly used $\hat{c}$ estimators for all models $\left\{\left(\phi^{G},p^{G}\right),\left(\phi^{G},p\right),\left(\phi ,p^{G}\right),\left(\phi ,p\right)\right\}$ in which γ=ρ=1. Dashed line at the value of $\hat{c}=1$

## Data Availability

The results in this body of work can be reproduced using the R code available in the following GitHub repository: https://github.com/AMDraghici/correlation_within_pair_bonds_CJS. The repository is archived with Zenodo (https://doi.org/10.5281/zenodo.4445346).

## APPENDIX A

### DERIVATIONS

Consider a fixed pair j∈{1,⋯,m} at fixed time t∈{1,⋯,T}. We provide derivations for the joint survival distribution, and we note that the results apply in general to the joint Bernoulli distribution under the presence of linear correlation. We use the notation and variables defined in Section 2.1 of the main document.

Joint distribution for survival and recapture processes

By definition the correlation coefficient of survival from time t‐1 to t between the individuals of pair j, after conditioning on $d_{j,t‐1}$ (event that pair j is together from time t‐1 to t), can be expressed as:

$$\gamma_{j,t‐1}d_{j,t‐1}=\frac{E\left(Y_{j,t}^{M}Y_{j,t}^{F}|Y_{j,t‐1}^{M}=1,Y_{j,t‐1}^{F}=1,d_{j,t‐1}\right)‐\phi_{j,t‐1}^{M}\phi_{j,t‐1}^{F}}{\sigma_{\phi ,j,t‐1}^{F}\sigma_{\phi ,j,t‐1}^{M}}$$

which implies,

$$E\left(Y_{j,t}^{M}Y_{j,t}^{F}|Y_{j,t‐1}^{M}=1,Y_{j,t‐1}^{F}=1,d_{j,t‐1}\right)=d_{j,t‐1}\gamma_{j,t‐1}\sigma_{\phi ,j,t‐1}^{F}\sigma_{\phi ,j,t‐1}^{M}+\phi_{j,t‐1}^{M}\phi_{j,t‐1}^{F}$$

as $E\left(Y_{j,t}^{G}\right)=\phi_{j,t‐1}^{G}$ since $Y_{j,t}^{G}|(Y_{j,t‐1}=1)∼\mathrm{Bernoulli}(\phi_{j,t‐1}^{G})$ for the individual of sex G∈{M,F}. Moreover, $E\left(Y_{j,t}^{M}=1,Y_{j,t}^{F}=1|Y_{j,t‐1}^{M}=1,Y_{j,t‐1}^{F}=1\right)=P\left(Y_{j,t}^{M}=1,Y_{j,t}^{F}=1|Y_{j,t‐1}^{M}=1,Y_{j,t‐1}^{F}=1\right)=\Phi_{j,t‐1}^{\mathrm{mf}}$. Therefore, dropping the indices for readability, the probability that both individuals from pair j survive from t‐1 to t, given that they are alive, is $\Phi^{\mathrm{mf}}=d\gamma \sigma^{F}\sigma^{M}+\phi^{M}\phi^{F}$. The remaining terms in the distribution follow from $\Phi^{\mathrm{mf}}$. The probability of one partner (of sex G) surviving but not the other is equal to the probability that the partner of sex G survives less the probability that both individuals survive. Therefore, $\Phi^{G0}=\phi^{G}‐\Phi^{\mathrm{mf}};\forall G\in \left\{M,F\right\}$. Moreover, the probability that both partners die is the compliment of all the other probabilities $\Phi^{00}=1‐\Phi^{\mathrm{mf}}‐\Phi^{m0}‐\Phi^{f0}$. Finally, to account for the possibility of temporary independence we conditioned $E(Y_{j,t}^{M}Y_{j,t}^{F}|Y_{j,t‐1}^{M}=1,Y_{j,t‐1}^{F}=1)$ on the variable $d_{j,t}$, which equals to zero when a couple is temporarily separated and gives rise to the joint Bernoulli distribution with no correlation +.

#### Bounds for correlation coefficients γ and ρ

Note that in this section we omit the indices j and t‐1. The first restriction on the joint distribution of survival for two living individuals is that the sum of the distinct event probabilities equals to one. Since the event of death for both individuals is equal to one less the other probabilities, this restriction can be expressed as $\Phi^{\mathrm{mf}}+\Phi^{m0}+\Phi^{f0}\leq 1$. It is also necessary that each probability term lies between zero and one. Equivalently, $\phi^{G}\geq \Phi^{\mathrm{mf}}\geq 0$ for G∈{M,F}. Finally, by definition the correlation coefficient is bounded above by one and below by negative one (γ∈[‐1,1]). These restrictions can be expressed in terms of γ to determine its bounds. Assume that the pairs are mated at time t so that d=1.

First note that $\phi^{G}\geq \Phi^{\mathrm{mf}}$ implies that

$$\phi^{G}\geq \Phi^{\mathrm{mf}}=\gamma \sigma^{F}\sigma^{M}+\phi^{M}\phi^{F}⇐\gamma \leq \frac{\phi^{G}‐\phi^{M}\phi^{F}}{\sigma^{F}\sigma^{M}};\forall G\in \left\{M,F\right\}.$$

Now given that $\sigma^{G}=\sqrt{\phi^{G}\left(1‐\phi^{G}\right)}$

$$\gamma \leq \frac{\phi^{G}‐\phi^{M}\phi^{F}}{\sqrt{\phi^{F}\left(1‐\phi^{F}\right)}\sqrt{\phi^{M}\left(1‐\phi^{M}\right)}};\forall G\in \left\{M,F\right\}.$$

Then WLOG let G=F to get:

$$\gamma \leq \frac{\phi^{F}‐\phi^{M}\phi^{F}}{\sqrt{\phi^{F}\left(1‐\phi^{F}\right)}\sqrt{\phi^{M}\left(1‐\phi^{M}\right)}}$$

$$=\sqrt{\frac{\phi^{F}\left(1‐\phi^{M}\right)}{\left(1‐\phi^{F}\right)\phi^{M}}}$$

$$=\sqrt{\frac{\mathrm{odds}(\phi^{F})}{\mathrm{odds}(\phi^{M})}}$$

$$=\sqrt{\mathrm{OR}(\phi^{F},\phi^{M})}$$

in which $\mathrm{OR}(\phi^{F},\phi^{M})$ denotes the odds ratio. Similarly, if G=M then

$$\gamma \leq \sqrt{\mathrm{OR}(\phi^{M},\phi^{F})}$$

$$=\frac{1}{\sqrt{\mathrm{OR}(\phi^{F},\phi^{M})}}$$

Further, since $\Phi^{\mathrm{mf}}=\gamma \sigma^{F}\sigma^{M}+\phi^{M}\phi^{F}\geq 0$,

$$\gamma \geq ‐\frac{\phi^{M}\phi^{F}}{\sigma^{F}\sigma^{M}}$$

$$=‐\frac{\left({\sqrt{\phi^{M}}}^{2}\right)\left({\sqrt{\phi^{F}}}^{2}\right)}{\sqrt{\phi^{F}\left(1‐\phi^{F}\right)}\sqrt{\phi^{M}\left(1‐\phi^{M}\right)}}$$

$$=‐\sqrt{\frac{\phi^{M}\phi^{F}}{\left(1‐\phi^{F}\right)\left(1‐\phi^{M}\right)}}$$

$$=‐\sqrt{\left(\frac{\phi^{M}}{1‐\phi^{M}}\right)\left(\frac{\phi^{F}}{1‐\phi^{F}}\right)}$$

$$=‐\sqrt{\mathrm{odds}\left(\phi^{M}\right)\mathrm{odds}\left(\phi^{F}\right)}$$

$$=‐\sqrt{\mathrm{OP}(\phi^{F},\phi^{M})}.$$

in which the odds product is defined as OP(X,Y):=odds(X)odds(Y);∀X∈[0,1]&Y∈[0,1]

Finally, noting that $\Phi^{G0}=\phi^{G}‐\Phi^{\mathrm{mf}}$, the restriction $\Phi^{\mathrm{mf}}+\Phi^{m0}+\Phi^{f0}\leq 1$ can be expressed as

$$\phi^{M}+\phi^{F}‐\gamma \sigma^{F}\sigma^{M}‐\phi^{M}\phi^{F}\leq 1.$$

Hence,

$$\gamma \geq \frac{\phi^{M}+\phi^{F}‐\phi^{M}\phi^{F}‐1}{\sigma^{M}\sigma^{F}}$$

$$=\frac{\phi^{M}\left(1‐\phi^{F}\right)+\phi^{F}‐1}{\sqrt{\phi^{F}\left(1‐\phi^{F}\right)}\sqrt{\phi^{M}\left(1‐\phi^{M}\right)}}$$

$$=‐\frac{‐\phi^{M}\left(1‐\phi^{F}\right)+\left(1‐\phi^{F}\right)}{\sqrt{\phi^{F}\left(1‐\phi^{F}\right)}\sqrt{\phi^{M}\left(1‐\phi^{M}\right)}}$$

$$=‐\frac{\left(1‐\phi^{M}\right)\left(1‐\phi^{F}\right)}{\sqrt{\phi^{F}\left(1‐\phi^{F}\right)}\sqrt{\phi^{M}\left(1‐\phi^{M}\right)}}$$

$$=‐\sqrt{\frac{\left(1‐\phi^{M}\right)\left(1‐\phi^{F}\right)}{\phi^{F}\phi^{M}}}$$

$$=‐\frac{1}{\sqrt{\mathrm{OP}(\phi^{F},\phi^{M})}}.$$

Putting these together yields the correlation bounds for the joint Bernoulli distribution:

$$\gamma \in \left[‐\min\left(\frac{1}{\sqrt{\mathrm{OP}(\phi^{F},\phi^{M})}},\sqrt{\mathrm{OP}(\phi^{F},\phi^{M})}\right),\min\left(\frac{1}{\sqrt{\mathrm{OR}(\phi^{F},\phi^{M})}},\sqrt{\mathrm{OR}(\phi^{F},\phi^{M})}\right)\right]$$

## APPENDIX B

### EXAMPLES

#### Standard error estimates under pair‐specific linear correlation

In this section, we provide an example illustrating why failing to differentiate between survival probabilities for sex‐specific groupings in the CJS model will result in underestimated standard errors when the data contain correlation between mated pairs. Consider modeling a set of known‐fate data, a special case of CJS data in which there is known perfect detection. Specifically, if individuals are not spotted by the researchers at any given sampling occasion they must have emigrated or perished at some earlier time in the study period. Furthermore, define $M_{t}$ and $F_{t}$ as the number of males and females that are captured and released at time t. Under this simplified parameter space, the MLE of the survival from time t to t+1 is ${\hat{\phi }}_{t}=\frac{M_{t}+F_{t}}{M_{t‐1}+F_{t‐1}}$. If we further assume that we have a population of animals that consists only of mated pairs with perfect linear survival dependence (γ=1), then we have that $M_{t}=F_{t}$.

##### Part 1: Assessing the reduced model (ϕ,p)

Fitting the standard CJS model, we find that ${\hat{\phi }}_{t}=\frac{M_{t}+M_{t}}{M_{t‐1}+M_{t‐1}}=\frac{2M_{t}}{2M_{t‐1}}=\frac{M_{t}}{M_{t‐1}}$. The estimate of standard deviation becomes $\hat{SE({\hat{\phi }}_{t})}=\sqrt{\frac{{\hat{\phi }}_{t}\left(1‐{\hat{\phi }}_{t}\right)}{M_{t‐1}}}$ since the number of males that survive from time t to t+1 can be now modeled by a binomial distribution $\sum_{i=1}^{M_{t‐1}}Y_{i,t}|Y_{i,t‐1}∼\mathrm{Binomial}(M_{t‐1},\phi_{t}Y_{i,t‐1})$. Note that exactly the same calculation can be made with data from females since $M_{t}=F_{t}$. However, the standard error calculated under the assumption of independence would be $SE_{I}\left({\hat{\phi }}_{t}\right)=\sqrt{\frac{{\hat{\phi }}_{t}\left(1‐{\hat{\phi }}_{t}\right)}{M_{t‐1}+F_{t‐1}}}=\sqrt{\frac{{\hat{\phi }}_{t}\left(1‐{\hat{\phi }}_{t}\right)}{2M_{t‐1}}}\approx \frac{SE({\hat{\phi }}_{t})}{\sqrt{2}}$. Therefore, in this example, we have that the standard errors of our survival probability estimates are being understated by a factor of $\sqrt{2}$. Wald based confidence intervals will then be too narrow by a factor of $\sqrt{2}$. The coverage of a 95% confidence interval will be about 83%. This example corresponds to the case in which $\hat{c}=2$ It is worth noting that the normal approximation typically is not suitable for mark–recapture estimates due to the highly non‐normal variance structure along with the fact that the estimates typically need to lie between [0,1] (Lebreton et al., 1992). The typical approach is instead to construct a normally approximated interval around the logit transformation of the parameter estimate with the delta method and back‐transform using the expit transformation. This may dampen the effect if the standard error is large or the estimate is close to either 0 or 1 since this approach squeezes the interval around the end points (Lebreton et al., 1992).

##### Part 2: Assessing the sex‐specific model $\left(\phi^{G},p\right)$

Now consider the model in which survival is estimated separately for both males and females, denoted $\left(\phi^{G},p\right)$. Survival is then estimated as ${\hat{\phi }}_{t}^{M}=\frac{M_{t}}{M_{t‐1}}$and ${\hat{\phi }}_{t}^{F}=\frac{F_{t}}{F_{t‐1}}$for males and females, respectively. Furthermore, standard errors become $SE\left({\hat{\phi }}_{t}^{F}\right)=\sqrt{\frac{{\hat{\phi }}_{t}^{F}\left(1‐{\hat{\phi }}_{t}^{F}\right)}{F_{t‐1}}}$for females and $SE\left({\hat{\phi }}_{t}^{M}\right)=\sqrt{\frac{{\hat{\phi }}_{t}^{M}\left(1‐{\hat{\phi }}_{t}^{M}\right)}{M_{t‐1}}}$for males. Since our assumption of perfect linear survival correlation gives us that $M_{t}=F_{t};\forall t\in \{1,\cdots ,T\}$we get that $SE\left({\hat{\phi }}_{t}^{F}\right)\approx SE\left({\hat{\phi }}_{t}^{M}\right)$, which are both equal to the correct standard error given in Part One. As such, our coverage percentages are unaffected. The results shown here are similar when considering correlated recapture probabilities as well.

#### The likelihood ratio test under pair‐specific linear correlation

In this section, we compare the behavior of the deviance statistic for testing for an effect of sex on survival when the data either contain exact replicate capture histories or when there is sex‐specific correlation between survival and recapture outcomes of mated pairs. In Part One, we provide a mathematical example comparing the behavior of the deviance statistic (for the LRT of $\left(\phi^{G},p\right)$ against (ϕ,p)) for the case in which the mark–recapture data under study contain sex‐specific correlation between survival and recapture outcomes. In Part Two, we repeat the calculation in Part One but instead consider the case in which the data have replicates but no group‐specific correlation in either survival or recapture. Finally, in Part Three we simulate the distribution of both the deviance and its corresponding p‐values using mark–recapture data of size n=100 and n=200 to show the impact of halving the sample size of each dataset.

##### Part 1: Asymptotic behavior under perfect linear correlation

Consider the likelihood ratio test between the $\left(\phi^{G},p\right)$ and (ϕ,p) CJS models. Assume that both recapture and survival of males and females are perfectly correlated (which can only occur when $\phi^{F}=\phi^{M}$ and $p^{F}=p^{M}$, respectively) in a population of animals that are 50% male and female, with 100% of the members being mated. Furthermore, assume that there is no temporal variation in the survival and recapture probabilities. For convenience, we calculate the deviance for the case in which there is only one model cohort with first capture at t=1 (denote this as $A_{j}=1;\forall j$). Let n be the number of marked individuals within our population. Define $h_{j}$ to be the cell frequency of capture history j (there are $2^{T‐1}$ possible outcomes for this cohort). Let $\mu_{j}:=E\left(h_{j}\right)=nP\left(Z=j|A_{j}=1\right)$ be the expected cell frequency of capture history j in which Z=j denotes that capture history j occurred. Then, the multinomial log‐likelihood under the null hypothesis would be:

$${\mathrm{LL}}_{0}=\sum_{j=1}^{2^{T‐1}}h_{j}\mathrm{Log}\left(\mu_{j}/n\right).$$

Under the alternative hypothesis, the log‐likelihood becomes

$${\mathrm{LL}}_{\alpha }=\sum_{G\in \{M,F\}}\sum_{j=1}^{2^{T‐1}}h_{j}^{G}\mathrm{Log}(\mu_{j}^{G}/n^{G})$$

in which $h_{j}^{G}$ and $\mu_{j}^{G}$ are the observed and expected cell frequencies for capture history j for sexes G∈{M,F} and $n^{G}$ is the amount of marked individuals in sexes G∈{M,F}. Under this setup, $h_{j}^{G}=h_{j}/2$ given that each pair will have identical observed histories (perfectly correlated recapture and survival fates). Furthermore, the expected cell frequency of history j becomes $\mu_{j}^{G}=E\left(h_{j}^{G}\right)=n^{G}P\left(h_{j}^{G}=h_{j}^{G}|A_{j}=1\right)=\mu_{j}/2$ since $n^{G}=n/2$. Now we compute the deviance to get:

$$‐2\mathrm{Log}\left(\Delta \right)=‐2{\mathrm{LL}}_{0}‐\left(‐2{\mathrm{LL}}_{\alpha }\right)$$

$$=‐2\left(\sum_{j=1}^{2^{T‐1}}h_{j}\mathrm{Log}\left(\mu_{j}/n\right)‐\sum_{j=1}^{2^{T‐1}}h_{j}^{F}\mathrm{Log}\left(\mu_{j}^{F}/n^{F}\right)‐\sum_{j=1}^{2^{T‐1}}h_{j}^{M}\mathrm{Log}\left(\mu_{j}^{M}/n^{M}\right)\right)$$

$$=‐2\sum_{j=1}^{2^{T‐1}}\left(h_{j}\mathrm{Log}\left(\mu_{j}/n\right)‐\frac{h_{j}}{2}\mathrm{Log}\left(\frac{\mu_{j}/2}{n/2}\right)‐\frac{h_{j}}{2}\mathrm{Log}\left(\frac{\mu_{j}/2}{n/2}\right)\right)$$

$$=‐2\sum_{j=1}^{2^{T‐1}}\left(h_{j}\mathrm{Log}\left(\mu_{j}/n\right)‐h_{j}\mathrm{Log}\left(\mu_{j}/n\right)\right)$$

=0

Therefore, for a population consisting entirely of mated individuals with an equal number of males and females, we get that γ=1 and ρ=1 implies that ‐2Log(Δ)=0. As such, we can see that the extra‐binomial variation stemming from sex‐specific correlation deflates the likelihood ratio test statistic.

##### Part 2: Asymptotic behavior for replicated data without accounting for groups

Consider the setup from the previous example and now assume that there is no pair‐specific correlation present (γ=ρ=0). Further assume that we took our mark–recapture data and replicated all of the observed entries c times. Then, our new observed and expected cell frequencies are $h_{j}^{\mathrm{New}}=ch_{j}$ and $\mu_{j}^{\mathrm{New}}=n^{\mathrm{New}}P\left(Z=j|A_{j}=1\right)=cnP\left(Z=j|A_{j}=1\right)=c\mu_{j}$. The same relationships hold for sex‐specific cell frequencies as well. Then, the deviance statistic for the LRT between the models (ϕ,p) and $\left(\phi^{G},p\right)$ is computed as:

$$‐2\mathrm{Log}\left(\Delta^{\mathrm{New}}\right)=‐2{\mathrm{LL}}_{0}^{\mathrm{New}}‐\left(‐2{\mathrm{LL}}_{\alpha }^{\mathrm{New}}\right)$$

$$=‐2\left(\sum_{j=1}^{2^{T‐1}}ch_{j}\mathrm{Log}\left(c\mu_{j}/cn\right)‐\sum_{j=1}^{2^{T‐1}}ch_{j}^{F}\mathrm{Log}\left(c\mu_{j}^{F}/cn^{F}\right)‐\sum_{j=1}^{2^{T‐1}}ch_{j}^{M}\mathrm{Log}\left(c\mu_{j}^{M}/cn^{M}\right)\right)$$

$$=‐2\sum_{j=1}^{2^{T‐1}}\left(ch_{j}\mathrm{Log}\left(\mu_{j}/n\right)‐ch_{j}^{M}\mathrm{Log}\left(\mu_{j}^{M}/n^{M}\right)‐ch_{j}^{F}\mathrm{Log}\left(\mu_{j}^{F}/n^{F}\right)\right)$$

=c(‐2Log(Δ)).

Therefore, when dealing with replicated data, the deviance is equal to the deviance of one replicate multiplied by the number of replications.

##### Part 3: Effect of halving data without any linear correlation

In this example, we conduct a small simulation study on the likelihood ratio test between the models $\left(\phi^{G},p\right)$ and (ϕ,p) in order to determine whether the violations in section 3.2 of the main document might be due to spare count data. We assume that there is no correlation between males or females for recapture or survival outcomes. We generated 1000 iterations for both models and compute the density of the deviance statistic and the p‐value for the cases in which n=100 and n=200. Otherwise, the model settings are the same as outlined in section 2.2 of the main document. Consider the results in Figure 6—halving the sample size of the data does not result in the large violation of asymptotic behavior that we are observing when there are correlations introduced between mated pairs. As such, we can conclude that the violation of assumptions that we are seeing in section 3.2 are not due to sparse cell observations.

#### Estimating $\hat{c}$ under Pair‐specific linear correlation

In this section, we study the behavior of the deviance $\hat{c}$ estimator when mark–recapture data contain replicates against the case in which there is sex‐specific correlation. In Part One, we calculate the deviance $\hat{c}$ estimator for data in which there is perfect linear correlation in recapture and survival for mated pairs. In Part Two, we add to the mathematical result in Part One by computing the deviance $\hat{c}$ estimator for data in which there are perfect replicates. Finally, in Part Three we simulate the distribution of $\hat{c}$ for the three common estimators to illustrate that their computation is consistent with the results shown in our study.

##### Part 1: Computing $\hat{c}$ under Perfect Linear Correlation

Using the same notation as described in Appendix B2, the deviance statistic between the saturated model and the (ϕ,p) CJS model, for one model cohort at first capture $\left(A_{j}=1;\forall j\right)$, can be computed as:

$${\mathrm{Dev}}_{0}=‐2\sum_{j=1}^{2^{T‐1}}h_{j}\mathrm{Log}\left(\mu_{j}/h_{j}\right),$$

with degrees of freedom ${\mathrm{df}}_{0}=2^{T‐1}‐1‐n_{\mathrm{par}}=2^{T‐1}‐3$, since the number of parameters for model (ϕ,p) is $n_{\mathrm{par}}=2$.

Furthermore, the deviance between the saturated model and any of the following CJS models: $\left(\phi^{G},p\right)$, $\left(\phi ,p^{G}\right)$ and $\left(\phi^{G},p^{G}\right)$, for one cohort at first capture, can be computed as:

$${\mathrm{Dev}}_{G}=‐2\sum_{G\in \{M,F\}}\sum_{j=1}^{2^{T‐1}}h_{j}^{G}\mathrm{Log}(\mu_{j}^{G}/h_{j}^{G}),$$

with degrees of freedom ${\mathrm{df}}_{G}=2^{T}‐2‐n_{\mathrm{par}}$. Note that $n_{\mathrm{par}}$ is equal to three for models $\left(\phi^{G},p\right)$ and $\left(\phi ,p^{G}\right)$ and four for model $\left(\phi^{G},p^{G}\right)$.

Now, assume that both recapture and survival of males and females are perfectly correlated in a population of animals that are exactly 50% male and female with 100% of the members being mated. Furthermore, assume that there is no temporal variation in the survival and recapture probabilities. As shown in Appendix B2 we have that $h_{j}^{G}=h_{j}/2$, $\mu_{j}^{G}=\mu_{j}/2$ and $n^{G}=n/2$. Now we can plug these into ${\mathrm{Dev}}_{G}$ to get:

$${\mathrm{Dev}}_{G}=‐2\sum_{j=1}^{2^{T‐1}}\left(\frac{h_{j}}{2}\mathrm{Log}\left(\frac{\mu_{j}/2}{h_{j}/2}\right)+\frac{h_{j}}{2}\mathrm{Log}\left(\frac{\mu_{j}/2}{h_{j}/2}\right)\right)$$

$$=‐2\sum_{j=1}^{2^{T‐1}}h_{j}\mathrm{Log}\left(\mu_{j}/h_{j}\right)$$

$$={\mathrm{Dev}}_{0}.$$

However, we have that $df_{G}‐df_{0}=2^{T}‐2‐n_{\mathrm{par}}‐2^{T‐1}+3=2^{T‐1}+1‐n_{\mathrm{par}}$, and when $n_{\mathrm{par}}=4$, we get $df_{G}‐df_{0}=2^{T‐1}‐3=df_{0}$. Thus, $df_{G}=2df_{0}$ for model $\left(\phi^{G},p^{G}\right)$ and $df_{G}=2df_{0}+1$ for models $\left(\phi^{G},p\right)$ and $\left(\phi ,p^{G}\right)$.

Now the estimate of c, for model $\left(\phi^{G},p^{G}\right)$ is computed as:

$${\hat{c}}_{G}={\mathrm{Dev}}_{G}/{\mathrm{df}}_{G}$$

$$={\mathrm{Dev}}_{0}/2{\mathrm{df}}_{0}$$

$$={\hat{c}}_{0}/2$$

in which ${\hat{c}}_{0}$ is the variance inflation correction for model (ϕ,p). Similarly, ${\hat{c}}_{G}={\hat{c}}_{0}/\left(2{\mathrm{df}}_{0}+1\right)$ if we are looking at models $\left(\phi ,p^{G}\right)$ or $\left(\phi^{G},p\right)$. This explains why the more general models that account for the correlated sex groups have lowered $\hat{c}$ values compared to the simple model that treats survival and recapture the same for both males and females.

##### Part 2: Computing $\hat{c}$ for replicated data without accounting for groups

Consider the setup from the previous example and now assume that there is no pair‐specific correlation present (γ=ρ=0). Further assume that we took our mark–recapture data and replicated all of the observed entries c times. Then, our new observed and expected cell frequencies are $h_{j}^{\mathrm{New}}=ch_{j}$ and $\mu_{j}^{\mathrm{New}}=c\mu_{j}$. The same relationships hold for sex‐specific cell frequencies as well. Now the deviance statistic between the saturated model and the (ϕ,p) CJS model, for one model cohort at first capture $\left(A_{j}=1;\forall j\right)$ with the replicated data, can be computed as:

$${\mathrm{Dev}}_{0}^{\mathrm{New}}=‐2\sum_{j=1}^{2^{T‐1}}h_{j}^{\mathrm{New}}\mathrm{Log}\left(\mu_{j}^{\mathrm{New}}/h_{j}^{\mathrm{New}}\right)$$

$$=‐2\sum_{j=1}^{2^{T‐1}}ch_{j}\mathrm{Log}\left(c\mu_{j}/ch_{j}\right)$$

$$=c{\mathrm{Dev}}_{0},$$

with degrees of freedom ${\mathrm{df}}_{0}=2^{T‐1}‐1‐n_{\mathrm{par}}=2^{T‐1}‐3$, since the number of parameters for model (ϕ,p) is $n_{\mathrm{par}}=2$.

Furthermore, the deviance between the saturated model and any of the following CJS models: $\left(\phi^{G},p\right)$, $\left(\phi ,p^{G}\right)$ and $\left(\phi^{G},p^{G}\right)$, for one cohort at first capture with the replicated data, can be computed as:

$${\mathrm{Dev}}_{G}^{\mathrm{New}}=‐2\sum_{G\in \{M,F\}}\sum_{j=1}^{2^{T‐1}}h_{j}^{G,\mathrm{New}}\mathrm{Log}(\mu_{j}^{G,\mathrm{New}}/h_{j}^{G,\mathrm{New}})$$

$$=‐2\sum_{G\in \{M,F\}}\sum_{j=1}^{2^{T‐1}}ch_{j}^{G}\mathrm{Log}(c\mu_{j}^{G}/ch_{j}^{G})$$

$$=c{\mathrm{Dev}}_{G},$$

with degrees of freedom ${\mathrm{df}}_{G}=2^{T}‐2‐n_{\mathrm{par}}$. Note that $n_{\mathrm{par}}$ is equal to three for models $\left(\phi^{G},p\right)$ and $\left(\phi ,p^{G}\right)$ and four for model $\left(\phi^{G},p^{G}\right)$. Therefore, the deviance terms are equal to the deviance for a single replicate multiplied by the number of replicates. The degrees of freedom are not impacted by replicated data, so they remain unchanged. As such, the estimates $\hat{c}$ will be equal to the estimate of the overdispersion for one replicate (theoretically this is equal to one) multiplied by the number of replicates.

##### Part 3: Comparing Estimators of $\hat{c}$

In this section, we conduct a small simulation study to compare the different estimators of c. Assume we have identical parameters to the settings (defined in section 2.2 in the main document) in which we set γ=ρ=1. We compute the densities of the deviance $\hat{c}$ (Anderson et al., 1994), Pearson's $\hat{c}$ (Lebreton et al., 1992; Pradel et al., 2005), and Fletcher's $\hat{c}$ (Afroz et al., 2019; Fletcher, 2012) across all four models cases. Consider the results in Figure 7—we can see that the variance inflation factor based on Pearson's statistic and the one proposed by Fletcher both have nearly identical distributions when the dyads in the model are highly correlated. As expected, the deviance $\hat{c}$ statistic is biased high relative to the newer estimators as it has heavier tails (see Anderson et al., 1994 for instance). The increase in bias, however, does not impact the conclusions drawn from our study. As such, our findings hold regardless of which estimator of $\hat{c}$ is employed.

**TABLE 3:** Median($\hat{c}$) for common estimators across all models

	Estimator		
Model	Deviance	Pearson	Fletcher
(ϕ,p)	2.01	1.69	1.73
$\left(\phi ,p^{G}\right)$	0.95	0.80	0.81
$\left(\phi^{G},p\right)$	0.94	0.80	0.81
$\left(\phi^{G},p^{G}\right)$	1.04	0.88	0.88
