# Supplementary material for: Understanding the impact of correlation within pair‐bonds on Cormack–Jolly–Seber models
Source: Ecol Evol. 2021 May 1;11(11):5966–84. doi: 10.1002/ece3.7329 (PMC8207451; doi:10.1002/ece3.7329)
Supplement: Supplementary file 1 [file ECE3-11-5966-s001.pdf]

# A Appendix: Derivations

Consider a fixed pair $j \in \{1,\ldots,m\}$ at fixed time $t \in \{1,\ldots,T\}$. We provide derivations for the joint survival distribution and we note that the results apply in general to the joint Bernoulli distribution under the presence of linear correlation. We use the notation and variables defined in Section 2.1 of the main document.

## A.1 Joint Distribution for Survival and Recapture Processes

By definition the correlation coefficient of survival from time $t-1$ to $t$ between the individuals of pair $j$, after conditioning on $d_{j,t-1}$ (event that pair $j$ is together from time $t-1$ to $t$), can be expressed as:

$$\gamma_{j,t-1}d_{j,t-1} = \frac{E(Y^M_{j,t}Y^F_{j,t}|Y^M_{j,t-1}=1, Y^F_{j,t-1}=1, d_{j,t-1}) - \phi^M_{j,t-1}\phi^F_{j,t-1}}{\sigma^F_{\phi,j,t-1}\sigma^M_{\phi,j,t-1}}$$

which implies,

$$E(Y^M_{j,t}Y^F_{j,t}|Y^M_{j,t-1}=1, Y^F_{j,t-1}=1, d_{j,t-1}) = d_{j,t-1}\gamma_{j,t-1}\sigma^F_{\phi,j,t-1}\sigma^M_{\phi,j,t-1} + \phi^M_{j,t-1}\phi^F_{j,t-1}$$

as $E(Y^G_{j,t}) = \phi^G_{j,t-1}$ since $Y^G_{j,t}|(Y_{j,t-1}=1) \sim \text{Bernoulli}(\phi^G_{j,t-1})$ for the individual of sex $G \in \{M,F\}$. Moreover, $E(Y^M_{j,t}=1, Y^F_{j,t}=1|Y^M_{j,t-1}=1, Y^F_{j,t-1}=1) = \mathbb{P}(Y^M_{j,t}=1, Y^F_{j,t}=1|Y^M_{j,t-1}=1, Y^F_{j,t-1}=1) = \Phi^{mf}_{j,t-1}$. Therefore, dropping the indices for readability, the probability that both individuals from pair $j$ survive from $t-1$ to $t$, given that they are alive, is $\Phi^{mf} = d\gamma\sigma^F\sigma^M + \phi^M\phi^F$. The remaining terms in the distribution follow from $\Phi^{mf}$. The probability of one partner (of sex $G$) surviving but not the other is equal to the probability that the partner of sex $G$ survives less the probability that both individuals survive. Therefore $\Phi^{G0} = \phi^G - \Phi^{mf}; \forall G \in \{M,F\}$. Moreover, the probability that both partners die is the compliment of all the other probabilities $\Phi^{00} = 1 - \Phi^{mf} - \Phi^{m0} - \Phi^{f0}$. Finally, to account for the possibility of temporary independence we conditioned $E(Y^M_{j,t}Y^F_{j,t}|Y^M_{j,t-1}=1, Y^F_{j,t-1}=1)$ on the variable $d_{j,t}$, which equals to zero when a couple is temporarily separated and gives rise to the joint Bernoulli distribution with no correlation $\blacksquare$.

## A.2 Bounds for Correlation Coefficients $\gamma$ and $\rho$

Note that in this section we omit the indices $j$ and $t-1$. The first restriction on the joint distribution of survival for two living individuals is that the sum of the distinct event probabilities equals to one. Since the event of death for both individuals is equal to one less the other probabilities, this restriction can be expressed as $\Phi^{mf} + \Phi^{m0} + \Phi^{f0} \leq 1$. It is also necessary that each probability term lies between zero and one. Equivalently, $\phi^G \geq \Phi^{mf} \geq 0$ for $G \in \{M, F\}$. Finally, by definition the correlation coefficient is bounded above by one and below by negative one ($\gamma \in [-1, 1]$). These restrictions can be expressed in terms of $\gamma$ to determine its bounds. Assume that the pairs are mated at time $t$ so that $d = 1$.

First note that $\phi^G \geq \Phi^{mf}$ implies that

$$\phi^G \geq \Phi^{mf} = \gamma\sigma^F\sigma^M + \phi^M\phi^F \iff \gamma \leq \frac{\phi^G - \phi^M\phi^F}{\sigma^F\sigma^M}; \forall G \in \{M, F\}.$$

Now given that $\sigma^G = \sqrt{\phi^G(1-\phi^G)}$

$$\gamma \leq \frac{\phi^G - \phi^M\phi^F}{\sqrt{\phi^F(1-\phi^F)}\sqrt{\phi^M(1-\phi^M)}}; \forall G \in \{M, F\}.$$

Then WLOG let $G = F$ to get:

$$\begin{aligned}\gamma &\leq \frac{\phi^F - \phi^M\phi^F}{\sqrt{\phi^F(1-\phi^F)}\sqrt{\phi^M(1-\phi^M)}} \\ &= \sqrt{\frac{\phi^F(1-\phi^M)}{(1-\phi^F)\phi^M}} \\ &= \sqrt{\frac{\text{odds}(\phi^F)}{\text{odds}(\phi^M)}} \\ &= \sqrt{\text{OR}(\phi^F, \phi^M)}\end{aligned}$$

in which $\text{OR}(\phi^F, \phi^M)$ denotes the odds ratio. Similarly, if $G = M$ then

$$\begin{aligned}\gamma &\leq \sqrt{\mathrm{OR}(\phi^M, \phi^F)} \\ &= \frac{1}{\sqrt{\mathrm{OR}(\phi^F, \phi^M)}}\end{aligned}$$

Further, since $\Phi^{mf} = \gamma\sigma^F\sigma^M + \phi^M\phi^F \geq 0$,

$$\begin{aligned}\gamma &\geq -\frac{\phi^M\phi^F}{\sigma^F\sigma^M} \\ &= -\frac{(\sqrt{\phi^M})^2(\sqrt{\phi^F})^2}{\sqrt{\phi^F(1-\phi^F)}\sqrt{\phi^M(1-\phi^M)}} \\ &= -\sqrt{\frac{\phi^M\phi^F}{(1-\phi^F)(1-\phi^M)}} \\ &= -\sqrt{\left(\frac{\phi^M}{1-\phi^M}\right)\left(\frac{\phi^F}{1-\phi^F}\right)} \\ &= -\sqrt{\mathrm{odds}(\phi^M)\mathrm{odds}(\phi^F)} \\ &= -\sqrt{\mathrm{OP}(\phi^F, \phi^M)}.\end{aligned}$$

in which the odds product is defined as OP(X,Y) := odds(X)odds(Y); $\forall X \in [0,1] \& Y \in [0,1]$

Finally, noting that $\Phi^{G0} = \phi^G - \Phi^{mf}$, the restriction $\Phi^{mf} + \Phi^{m0} + \Phi^{f0} \leq 1$ can be expressed as

$$\phi^M + \phi^F - \gamma\sigma^F\sigma^M - \phi^M\phi^F \leq 1.$$

Hence,

$$
\begin{aligned}
\gamma &\geq \frac{\phi^M + \phi^F - \phi^M\phi^F - 1}{\sigma^M\sigma^F} \\
&= \frac{\phi^M(1-\phi^F) + \phi^F - 1}{\sqrt{\phi^F(1-\phi^F)}\sqrt{\phi^M(1-\phi^M)}} \\
&= -\frac{-\phi^M(1-\phi^F) + (1-\phi^F)}{\sqrt{\phi^F(1-\phi^F)}\sqrt{\phi^M(1-\phi^M)}} \\
&= -\frac{(1-\phi^M)(1-\phi^F)}{\sqrt{\phi^F(1-\phi^F)}\sqrt{\phi^M(1-\phi^M)}} \\
&= -\sqrt{\frac{(1-\phi^M)(1-\phi^F)}{\phi^F\phi^M}} \\
&= -\frac{1}{\sqrt{\mathrm{OP}(\phi^F,\phi^M)}}.
\end{aligned}
$$

Putting these together yields the correlation bounds for the joint Bernoulli distribution:

$$
\gamma \in \left[-\min\left(\frac{1}{\sqrt{\mathrm{OP}(\phi^F,\phi^M)}}, \sqrt{\mathrm{OP}(\phi^F,\phi^M)}\right), \min\left(\frac{1}{\sqrt{\mathrm{OR}(\phi^F,\phi^M)}}, \sqrt{\mathrm{OR}(\phi^F,\phi^M)}\right)\right] \blacksquare
$$

# B Appendix: Examples

## B.1 Standard Error Estimates under Pair-Specific Linear Correlation

In this section, we provide an example illustrating why failing to differentiate between survival probabilities for sex-specific groupings in the CJS model will result in underestimated standard errors when the data contains correlation between mated pairs. Consider modelling a set of known-fate data, a special case of CJS data in which there is known perfect detection. Specifically, if individuals are not spotted by the researchers at any given sampling occasion they must have emigrated or perished at some earlier time in the study period. Furthermore, define $M_t$ and $F_t$ as the number of males and females that are captured and released at time $t$. Under this simplified parameter space, the MLE of the survival from time $t$ to $t+1$ is $\hat{\phi}_t = \frac{M_t+F_t}{M_{t-1}+F_{t-1}}$. If we further assume that we have a population of animals that consists only of mated pairs with perfect linear survival dependence ($\gamma = 1$), then we have that $M_t = F_t$.

**Part 1: Assessing the Reduced Model** $(\phi, p)$

Fitting the standard CJS model we find that $\hat{\phi}_t = \frac{M_t+M_t}{M_{t-1}+M_{t-1}} = \frac{2M_t}{2M_{t-1}} = \frac{M_t}{M_{t-1}}$. The estimate of standard deviation becomes $\widehat{SE(\hat{\phi}_t)} = \sqrt{\frac{\hat{\phi}_t(1-\hat{\phi}_t)}{M_{t-1}}}$ since the number of males that survive from time $t$ to $t+1$ can be now modelled by a binomial distribution $\sum_{i=1}^{M_{t-1}} Y_{i,t}|Y_{i,t-1} \sim \text{Binomial}(M_{t-1}, \phi_t Y_{i,t-1})$. Note that exactly the same calculation can be made with data from females since $M_t = F_t$. However, the standard error calculated under the assumption of independence would be $SE_I(\hat{\phi}_t) = \sqrt{\frac{\hat{\phi}_t(1-\hat{\phi}_t)}{M_{t-1}+F_{t-1}}} = \sqrt{\frac{\hat{\phi}_t(1-\hat{\phi}_t)}{2M_{t-1}}} \approx \frac{SE(\hat{\phi}_t)}{\sqrt{2}}$. Therefore, in this example, we have that the standard errors of our survival probability estimates are being understated by a factor of $\sqrt{2}$. Wald based confidence intervals will then be too narrow by a factor of $\sqrt{2}$. The coverage of a 95% confidence interval will be about 83%. This example corresponds to the case in which $\hat{c} = 2$. It is worth noting that the normal approximation typically is not suitable for mark-recapture estimates due to the highly non-normal variance structure along with the fact that the estimates typically need to lie between $[0, 1]$ (Lebreton et al., 1992). The typical approach is instead to construct a normally approximated interval around the logit transformation of the parameter estimate with the delta method and back-transform using the expit transformation. This may dampen the effect if the standard error is large or the estimate is close to either 0 or 1 since this approach squeezes the interval around the end points (Lebreton et al., 1992).

**Part Two: Assessing the Sex-Specific Model** $(\phi^G, p)$

Now consider the model in which survival is estimated separately for both males and females, denoted $(\phi^G, p)$. Survival is then estimated as $\hat{\phi_t^M} = \frac{M_t}{M_{t-1}}$ and $\hat{\phi_t^F} = \frac{F_t}{F_{t-1}}$ for males and females, respectively.

Furthermore, standard errors become $SE(\hat{\phi_t^F}) = \sqrt{\frac{\hat{\phi_t^F}(1-\hat{\phi_t^F})}{F_{t-1}}}$ for females and $SE(\hat{\phi_t^M}) = \sqrt{\frac{\hat{\phi_t^M}(1-\hat{\phi_t^M})}{M_{t-1}}}$ for males. Since our assumption of perfect linear survival correlation gives us that $M_t = F_t;\ \forall t \in \{1, \ldots, T\}$ we get that $SE(\hat{\phi_t^F}) \approx SE(\hat{\phi_t^M})$, which are both equal to the correct standard error given in Part One. As such, our coverage percentages are unaffected. The results shown here are similar when considering correlated recapture probabilities as well. ■

## B.2 The Likelihood Ratio Test under Pair-Specific Linear Correlation

In this section, we compare the behaviour of the deviance statistic for testing for an effect of sex on survival when the data either contains exact replicate capture histories or when there is sex-specific correlation between survival and recapture outcomes of mated pairs. In Part One we provide a mathematical example comparing the behaviour of the deviance statistic (for the LRT of $(\phi^G, p)$ against $(\phi, p)$) for the case in which the mark-recapture data under study contains sex-specific correlation between survival and recapture outcomes. In Part Two we repeat the calculation in Part One but instead consider the case in which the data has replicates but no group-specific correlation in either survival or recapture. Finally, in Part Three we simulate the distribution of both the deviance and its corresponding $p$-values using mark-recapture data of size $n = 100$ and $n = 200$ to show the impact of halving the sample size of each dataset.

**Part One: Asymptotic Behaviour under Perfect Linear Correlation**

Consider the likelihood ratio test between the $(\phi^G, p)$ and $(\phi, p)$ CJS models. Assume that both recapture and survival of males and females is perfectly correlated (which can only occur when $\phi^F = \phi^M$ and $p^F = p^M$, respectively) in a population of animals that are 50% male and female, with 100% of the members being mated. Furthermore, assume that there is no temporal variation in the survival and recapture probabilities. For convenience, we calculate the deviance for the case in which there is only one model cohort with first capture at $t = 1$ (denote this as $A_j = 1; \forall j$). Let $n$ be the number of marked individuals within our population. Define $h_j$ to be the cell frequency of capture history $j$ (there are $2^{T-1}$ possible outcomes for this cohort). Let $\mu_j := \mathbb{E}(h_j) = n\mathbb{P}(Z = j|A_j = 1)$ be the expected cell frequency of capture history $j$ in which $Z = j$ denotes that capture history $j$ occurred. Then the multinomial log-likelihood under the null hypothesis would be:

$$\text{LL}_0 = \sum_{j=1}^{2^{T-1}} h_j \text{Log}(\mu_j/n).$$

Under the alternative hypothesis the log-likelihood becomes

$$\mathrm{LL}_\alpha = \sum_{G\in\{M,F\}} \sum_{j=1}^{2^{T-1}} h_j^G \mathrm{Log}(\mu_j^G/n^G)$$

in which $h_j^G$ and $\mu_j^G$ are the observed and expected cell frequencies for capture history $j$ for sexes $G \in \{M,F\}$ and $n^G$ is the amount of marked individuals in sexes $G \in \{M,F\}$. Under this setup $h_j^G = h_j/2$ given that each pair will have identical observed histories (perfectly correlated recapture and survival fates). Furthermore, the expected cell frequency of history $j$ becomes $\mu_j^G = E(h_j^G) = n^G\mathbb{P}(h_j^G = h_j^G|A_j = 1) = \mu_j/2$ since $n^G = n/2$. Now we compute the deviance to get:

$$\begin{aligned}
-2\mathrm{Log}(\Delta) &= -2\mathrm{LL}_0 - (-2\mathrm{LL}_\alpha)\\
&= -2\left(\sum_{j=1}^{2^{T-1}} h_j\mathrm{Log}(\mu_j/n) - \sum_{j=1}^{2^{T-1}} h_j^F\mathrm{Log}(\mu_j^F/n^F) - \sum_{j=1}^{2^{T-1}} h_j^M\mathrm{Log}(\mu_j^M/n^M)\right)\\
&= -2\sum_{j=1}^{2^{T-1}}\left(h_j\mathrm{Log}(\mu_j/n) - \frac{h_j}{2}\mathrm{Log}\left(\frac{\mu_j/2}{n/2}\right) - \frac{h_j}{2}\mathrm{Log}\left(\frac{\mu_j/2}{n/2}\right)\right)\\
&= -2\sum_{j=1}^{2^{T-1}}\left(h_j\mathrm{Log}(\mu_j/n) - h_j\mathrm{Log}\left(\mu_j/n\right)\right)\\
&= 0
\end{aligned}$$

Therefore, for a population consisting entirely of mated individuals with an equal number of males and females, we get that $\gamma = 1$ and $\rho = 1$ implies that $-2\mathrm{Log}(\Delta) = 0$. As such, we can see that the extra-binomial variation stemming from sex-specific correlation deflates the likelihood ratio test statistic.

**Part Two: Asymptotic Behaviour for Replicated Data without Accounting for Groups**

Consider the set up from the previous example and now assume that there is no pair-specific correlation present ($\gamma = \rho = 0$). Further assume that we took our mark-recapture data and replicated all of the observed entries $c$ times. Then our new observed and expected cell frequencies are $h_j^{\mathrm{New}} = ch_j$ and $\mu_j^{\mathrm{New}} = n^{\mathrm{New}}\mathbb{P}(Z = j|A_j = 1) = cn\mathbb{P}(Z = j|A_j = 1) = c\mu_j$. The same relationships hold for sex-specific cell frequencies as well. Then the deviance statistic for the LRT between the models $(\phi, p)$ and $(\phi^G, p)$ is computed as:

$$
\begin{aligned}
-2\text{Log}(\Delta)^{\text{New}} &= -2\text{LL}_0^{\text{New}} - (-2\text{LL}_\alpha)^{\text{New}} \\
&= -2\left(\sum_{j=1}^{2^{T-1}} ch_j \text{Log}(c\mu_j/cn) - \sum_{j=1}^{2^{T-1}} ch_j^F \text{Log}(c\mu_j^F/cn^F) - \sum_{j=1}^{2^{T-1}} ch_j^M \text{Log}(c\mu_j^M/cn^M)\right) \\
&= -2\sum_{j=1}^{2^{T-1}} \left(ch_j \text{Log}(\mu_j/n) - ch_j^M \text{Log}\left(\mu_j^M/n^M\right) - ch_j^F \text{Log}\left(\mu_j^F/n^F\right)\right) \\
&= c(-2\text{Log}(\Delta))\blacksquare
\end{aligned}
$$

Therefore, when dealing with replicated data, the deviance is equal to the deviance of one replicate multiplied by the number of replications.

**Part Three: Effect of Halving Data without any Linear Correlation**

In this example we conduct a small simulation study on the likelihood ratio test between the models $(\phi^G, p)$ and $(\phi, p)$ in order to determine whether the violations in section 3.2 of the main document might be due to spare count data. We assume that there is no correlation between males or females for recapture or survival outcomes. We generated 1000 iterations for both models and compute the density of the deviance statistic and the $p$-value for the cases in which $n = 100$ and $n = 200$. Otherwise, the model settings are the same as outlined in section 2.2 of the main document. Consider the results in Figure 6 - halving the sample size of the data does not result in the large violation of asymptotic behaviour that we are observing when there are correlations introduced between mated pairs. As such, we can conclude that the violation of assumptions that we are seeing in section 3.2 are not due to sparse cell observations.

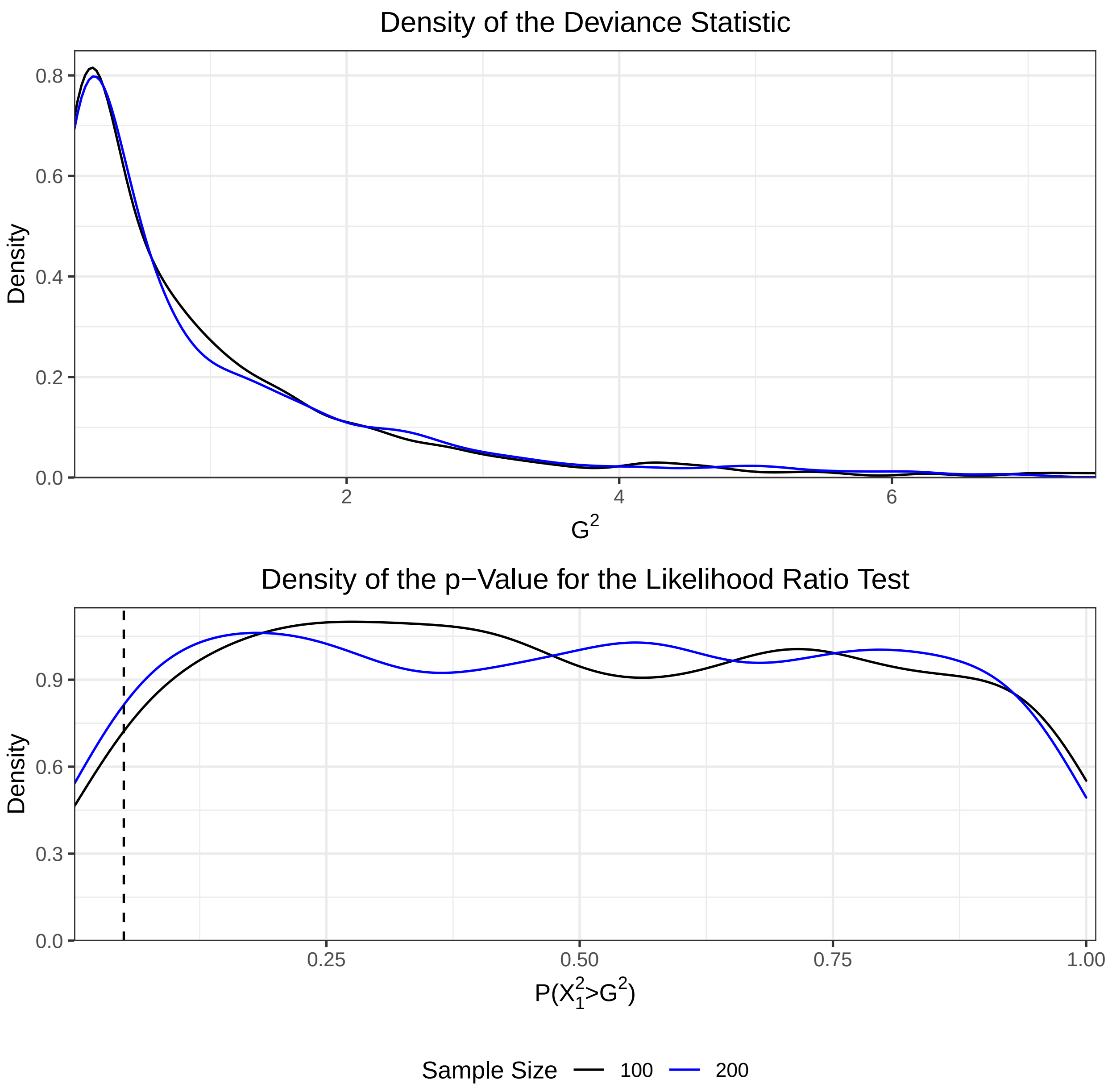


Figure 6: Density of the deviance and the $p$-values of the likelihood ratio test for $(\phi^G, p)$ vs $(\phi, p)$ in which $\rho = 0$ and $\gamma = 0$ for both n=100 and n=200. Dashed line at the value of $\mathbb{P}(\mathrm{X}_1^2 \geq \mathrm{G}^2) = 0.05$.

## B.3 Estimating $\hat{c}$ under Pair-Specific Linear Correlation

In this section, we study the behaviour of the deviance $\hat{c}$ estimator when mark-recapture data contains replicates against the case in which there is sex-specific correlation. In Part One we calculate the deviance $\hat{c}$ estimator for data in which there is perfect linear correlation in recapture and survival for mated pairs. In Part Two we add to the mathematical result in Part One by computing the deviance $\hat{c}$ estimator for data in which there are perfect replicates. Finally, in Part Three we simulate the distribution of $\hat{c}$ for the three common estimators to illustrate that their computation is consistent with the results shown in our study.

**Part One: Computing $\hat{c}$ under Perfect Linear Correlation**

Using the same notation as described in Appendix B.2, the deviance statistic between the saturated model and the $(\phi, p)$ CJS model, for one model cohort at first capture $(A_j = 1; \forall j)$, can be computed as:

$$\text{Dev}_0 = -2 \sum_{j=1}^{2^{T-1}} h_j \text{Log}(\mu_j / h_j),$$

with degrees of freedom $\text{df}_0 = 2^{T-1} - 1 - n_{\text{par}} = 2^{T-1} - 3$, since the number of parameters for model $(\phi, p)$ is $n_{\text{par}} = 2$.

Furthermore, the deviance between the saturated model and any of the following CJS models: $(\phi^G, p)$, $(\phi, p^G)$ and $(\phi^G, p^G)$, for one cohort at first capture, can be computed as:

$$\text{Dev}_G = -2 \sum_{G \in \{M,F\}} \sum_{j=1}^{2^{T-1}} h_j^G \text{Log}(\mu_j^G / h_j^G),$$

with degrees of freedom $\text{df}_G = 2^T - 2 - n_{\text{par}}$. Note that $n_{\text{par}}$ is equal to three for models $(\phi^G, p)$ and $(\phi, p^G)$ and four for model $(\phi^G, p^G)$.

Now, assume that both recapture and survival of males and females is perfectly correlated in a population of animals that are exactly 50% male and female with 100% of the members being mated. Furthermore, assume that there is no temporal variation in the survival and recapture probabilities. As shown in Appendix B.2 we have that $h_j^G = h_j/2$, $\mu_j^G = \mu_j/2$ and $n^G = n/2$. Now we can plug these into $\text{Dev}_G$ to get:

$$
\begin{aligned}
\mathrm{Dev}_G &= -2\sum_{j=1}^{2^{T-1}}\left(\frac{h_j}{2}\mathrm{Log}\left(\frac{\mu_j/2}{h_j/2}\right)+\frac{h_j}{2}\mathrm{Log}\left(\frac{\mu_j/2}{h_j/2}\right)\right)\\
&= -2\sum_{j=1}^{2^{T-1}} h_j\mathrm{Log}\left(\mu_j/h_j\right)\\
&= \mathrm{Dev}_0.
\end{aligned}
$$

However, we have that $df_G - df_0 = 2^T - 2 - n_{\text{par}} - 2^{T-1} + 3 = 2^{T-1} + 1 - n_{\text{par}}$ and when $n_{\text{par}} = 4$ we get $df_G - df_0 = 2^{T-1} - 3 = df_0$. Thus $df_G = 2df_0$ for model $(\phi^G, p^G)$ and $df_G = 2df_0 + 1$ for models $(\phi^G, p)$ and $(\phi, p^G)$.

Now the estimate of $c$, for model $(\phi^G, p^G)$ is computed as:

$$
\begin{aligned}
\hat{c}_G &= \mathrm{Dev}_G/\mathrm{df}_G\\
&= \mathrm{Dev}_0/2\mathrm{df}_0\\
&= \hat{c}_0/2
\end{aligned}
$$

in which $\hat{c}_0$ is the variance inflation correction for model $(\phi, p)$. Similarly, $\hat{c}_G = \hat{c}_0/(2\mathrm{df}_0 + 1)$ if we are looking at models $(\phi, p^G)$ or $(\phi^G, p)$. This explains why the more general models that account for the correlated sex-groups have lowered $\hat{c}$ values compared to the simple model that treats survival and recapture the same for both males and females ■

**Part Two: Computing $\hat{c}$ for Replicated Data without Accounting for Groups**

Consider the setup from the previous example and now assume that there is no pair-specific correlation present ($\gamma = \rho = 0$). Further assume that we took our mark-recapture data and replicated all of the observed entries $c$ times. Then our new observed and expected cell frequencies are $h_j^{\text{New}} = ch_j$ and $\mu_j^{\text{New}} = c\mu_j$. The same relationships hold for sex-specific cell frequencies as well. Now the deviance statistic between the saturated model and the $(\phi, p)$ CJS model, for one model cohort at first capture ($A_j = 1; \forall j$) with the replicated data, can be computed as:

$$
\begin{aligned}
\mathrm{Dev}_0^{\mathrm{New}} &= -2\sum_{j=1}^{2^{T-1}} h_j^{\mathrm{New}}\mathrm{Log}(\mu_j^{\mathrm{New}}/h_j^{\mathrm{New}}) \\
&= -2\sum_{j=1}^{2^{T-1}} ch_j\mathrm{Log}(c\mu_j/ch_j) \\
&= c\mathrm{Dev}_0,
\end{aligned}
$$

with degrees of freedom $\mathrm{df}_0 = 2^{T-1} - 1 - n_{\mathrm{par}} = 2^{T-1} - 3$, since the number of parameters for model $(\phi, p)$ is $n_{\mathrm{par}} = 2$.

Furthermore, the deviance between the saturated model and any of the following CJS models: $(\phi^G, p)$, $(\phi, p^G)$ and $(\phi^G, p^G)$, for one cohort at first capture with the replicated data, can be computed as:

$$
\begin{aligned}
\mathrm{Dev}_G^{\mathrm{New}} &= -2\sum_{G\in\{M,F\}}\sum_{j=1}^{2^{T-1}} h_j^{G,\mathrm{New}}\mathrm{Log}(\mu_j^{G,\mathrm{New}}/h_j^{G,\mathrm{New}}) \\
&= -2\sum_{G\in\{M,F\}}\sum_{j=1}^{2^{T-1}} ch_j^G\mathrm{Log}(c\mu_j^G/ch_j^G) \\
&= c\mathrm{Dev}_G,
\end{aligned}
$$

with degrees of freedom $\mathrm{df}_G = 2^T - 2 - n_{\mathrm{par}}$. Note that $n_{\mathrm{par}}$ is equal to three for models $(\phi^G, p)$ and $(\phi, p^G)$ and four for model $(\phi^G, p^G)$. Therefore, the deviance terms are equal to the deviance for a single replicate multiplied by the number of replicates. The degrees of freedom are not impacted by replicated data so they remain unchanged. As such, the estimates $\hat{c}$ will be equal to the estimate of the overdispersion for one replicate (theoretically this is equal to one) multiplied by the number of replicates ■

**Part Three: Comparing Estimators of $\hat{c}$**

In this section, we conduct a small simulation study to compare the different estimators of $c$. Assume we have identical parameters to the settings (defined in section 2.2 in the main document) in which we set $\gamma = \rho = 1$. We compute the densities of the deviance $\hat{c}$ (Anderson et al., 1994), Pearson's $\hat{c}$ (Lebreton et al., 1992; Pradel et al., 2005), and Fletcher's $\hat{c}$ (Fletcher, 2012; Afroz et al., 2019) across all four models cases.

Consider the results in Figure 7 - we can see that the variance inflation factor based on Pearson's statistic and the one proposed by Fletcher both have nearly identical distributions when the dyads in the model are highly correlated. As expected, the deviance $\hat{c}$ statistic is biased high relative to the newer estimators as it has heavier tails (see Anderson et al., 1994 for instance). The increase in bias, however, does not impact the conclusions drawn from our study. As such, our findings hold regardless of which estimator of $\hat{c}$ is employed.

Table 3: Median($\hat{c}$) for common estimators across all models

| | Estimator | | |
|---|---|---|---|
| Model | Deviance | Pearson | Fletcher |
| $(\phi, p)$ | 2.01 | 1.69 | 1.73 |
| $(\phi, p^G)$ | 0.95 | 0.80 | 0.81 |
| $(\phi^G, p)$ | 0.94 | 0.80 | 0.81 |
| $(\phi^G, p^G)$ | 1.04 | 0.88 | 0.88 |

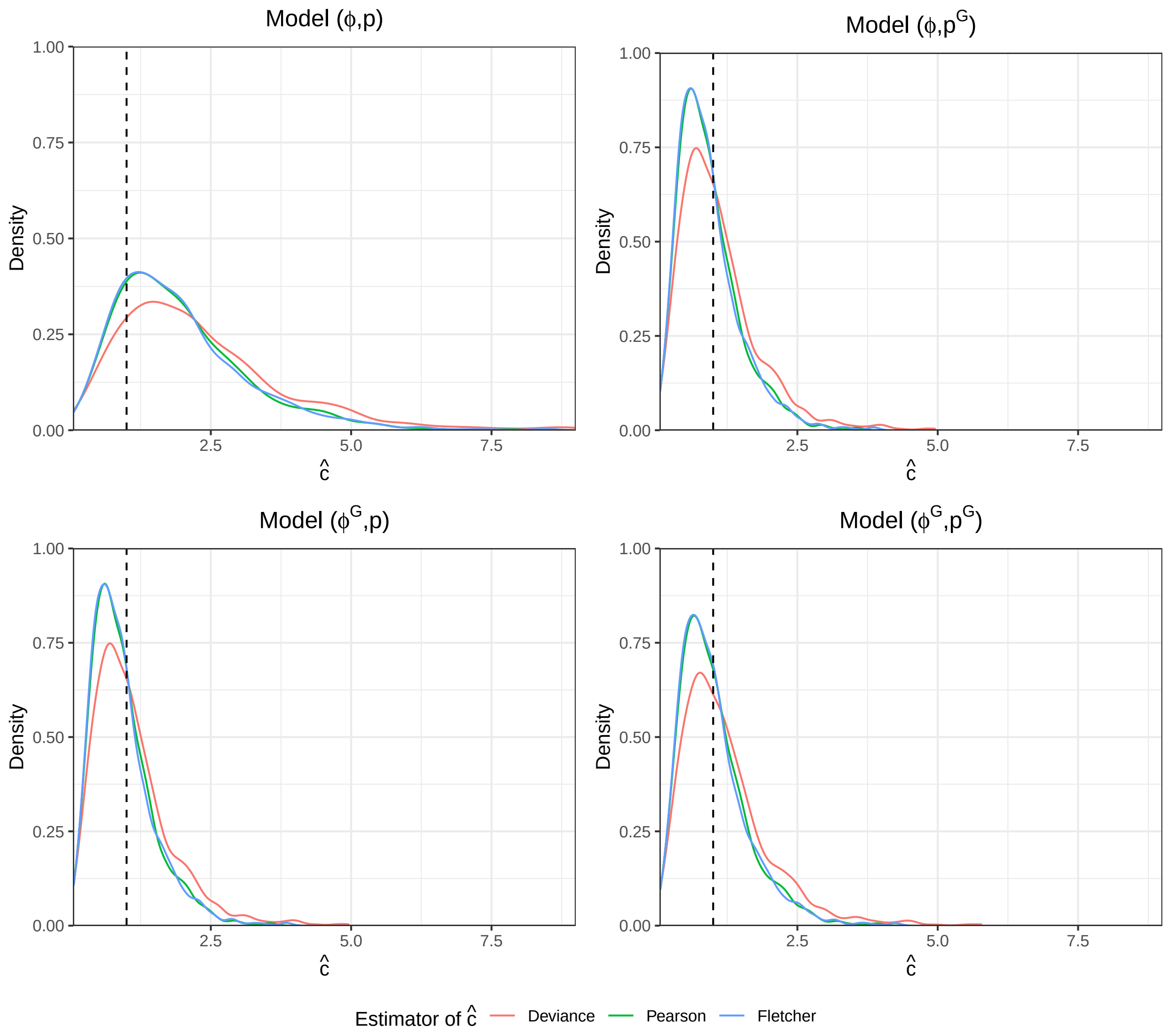


Figure 7: Density of commonly used $\hat{c}$ estimators for all models $\{(\phi^G, p^G), (\phi^G, p), (\phi, p^G), (\phi, p)\}$ in which $\gamma = \rho = 1$. Dashed line at the value of $\hat{c} = 1$.
